# The DNA replication checkpoint prevents PCNA/RFC depletion to protect forks from HLTF-induced collapse in human cells

**DOI:** 10.1016/j.molcel.2025.06.002

**Published:** 2025-06-26

**Authors:** Agostina P. Bertolin, Berta Canal, Mona Yekezare, Anne Early, Jingkun Zeng, Rachael Instrell, Michael Howell, John F.X. Diffley

**Affiliations:** 1 Chromosome Replication Laboratory, The Francis Crick Institute, London NW1 1AT, UK; 2 High Throughput Screening Laboratory, The Francis Crick Institute, London NW1 1AT, UK

## Abstract

The DNA replication checkpoint is crucial for maintaining genome stability after genotoxic stress; without it, stalled DNA replication forks cannot restart normally, excess DNA replication origins are activated, DNA damage and single-stranded DNA (ssDNA) accumulate, S phase does not finish, and cells die. Preventing excess origin firing suppresses all these effects. Here, we show in human cells that when replication is not restrained by a functional checkpoint, excess DNA synthesis sequesters the processivity factor PCNA and its loader, replication factor C (RFC), preventing normal fork restart. Nascent DNA ends unprotected by RFC/PCNA are attacked by the helicase-like transcription factor (HLTF), causing irreversible replication fork collapse and hyperaccumulation of ssDNA. This explains how the checkpoint stabilizes stalled replication forks and has implications for how origin firing is normally coordinated with fork progression. Loss of HLTF suppresses fork collapse and cell lethality in checkpoint-deficient cells, which has implications for how resistance to anti-checkpoint therapies may arise.

## Graphical abstract

**Graphical abstract:**
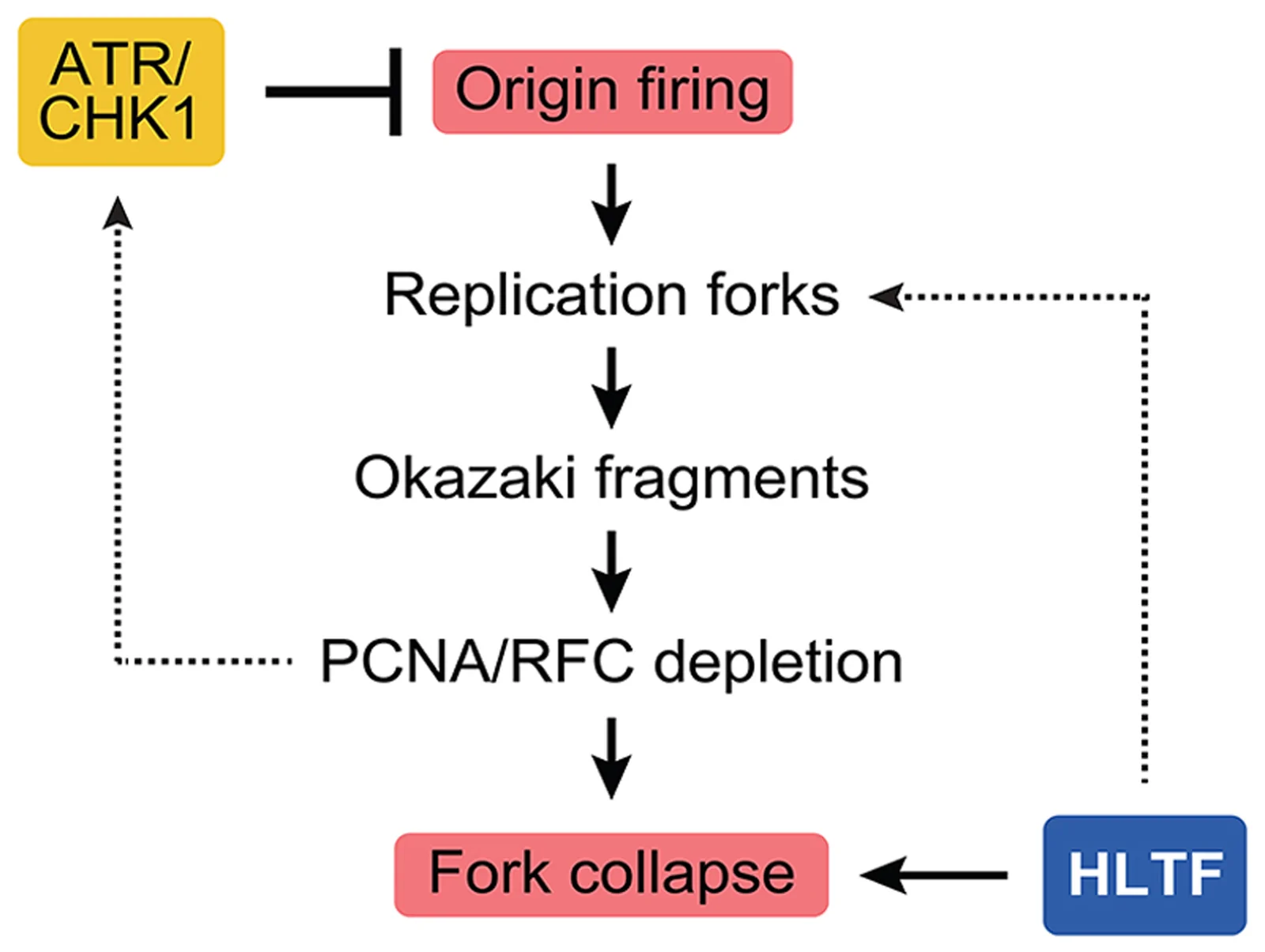

## Introduction

DNA replication fork stalling in response to nucleotide depletion, inhibition of DNA polymerases, or DNA damage activates the checkpoint protein kinases ATR and its downstream effector Chk1.^1–3^ In humans, mice, and budding yeast, these damage checkpoint kinases are essential for cell viability,^1,4–12^ and checkpoint defects in humans are associated with inherited developmental diseases, premature aging, and predisposition to various types of cancer.^13–18^ These kinases coordinate a wide range of cellular responses to promote genome stability and survival,^19^ including regulation of damage-dependent transcription, DNA repair, and cell-cycle arrest; however, the DNA-replication-associated functions of the checkpoint appear to be especially important.^20–22^ The checkpoint inhibits replication origin firing in yeast and humans; in yeast by inhibiting two replication origin firing factors, Sld3 and Dbf4,^23,24^ and in humans by targeting CDC25A, the CDK2 phosphatase and activator, for ubiquitin-mediated proteolysis.^25–27^

The checkpoint also protects stalled replication forks in yeast and humans from undergoing catastrophic changes often referred to as ‘‘fork collapse.’’^28^ Replisomes stall irreversibly and can no longer resume replication, even if the checkpoint is restored,^29,30^ and normal DNA replication fork structures are converted into structures associated with recombination,^30^ despite the fact that CMG helicase and associated factors remain associated with DNA.^31^ How checkpoints stabilize replication forks and the mechanistic basis for irreversible fork arrest are currently poorly understood. Nucleases including Exo1,^32–34^ MRX,^35^ and the proofreading exonuclease of DNA polymerase ε^36^ have all been implicated in this phenomenon in yeast. Evidence from human cells indicates that deregulation of origin firing in the absence of a functional checkpoint drives fork collapse^28^ and that accumulation of single-stranded DNA (ssDNA) during this process depletes the soluble pool of the ssDNA-binding protein RPA, which causes DNA damage.^37^ In this manuscript, we have re-examined events occurring at stalled replication forks in the absence of a functional checkpoint in human cells, and we provide evidence that it is depletion of replication factor C (RFC), the PCNA sliding clamp loader, by excess Okazaki fragments (OkFs) that is the initial driver of fork collapse and that the Snf2-family DNA translocase helicase-like transcription factor (HLTF) executes the irreversible fork collapse.

## Results

### Checkpoint inhibition after replication fork stalling induces S-phase catastrophe

**Figure 1:**
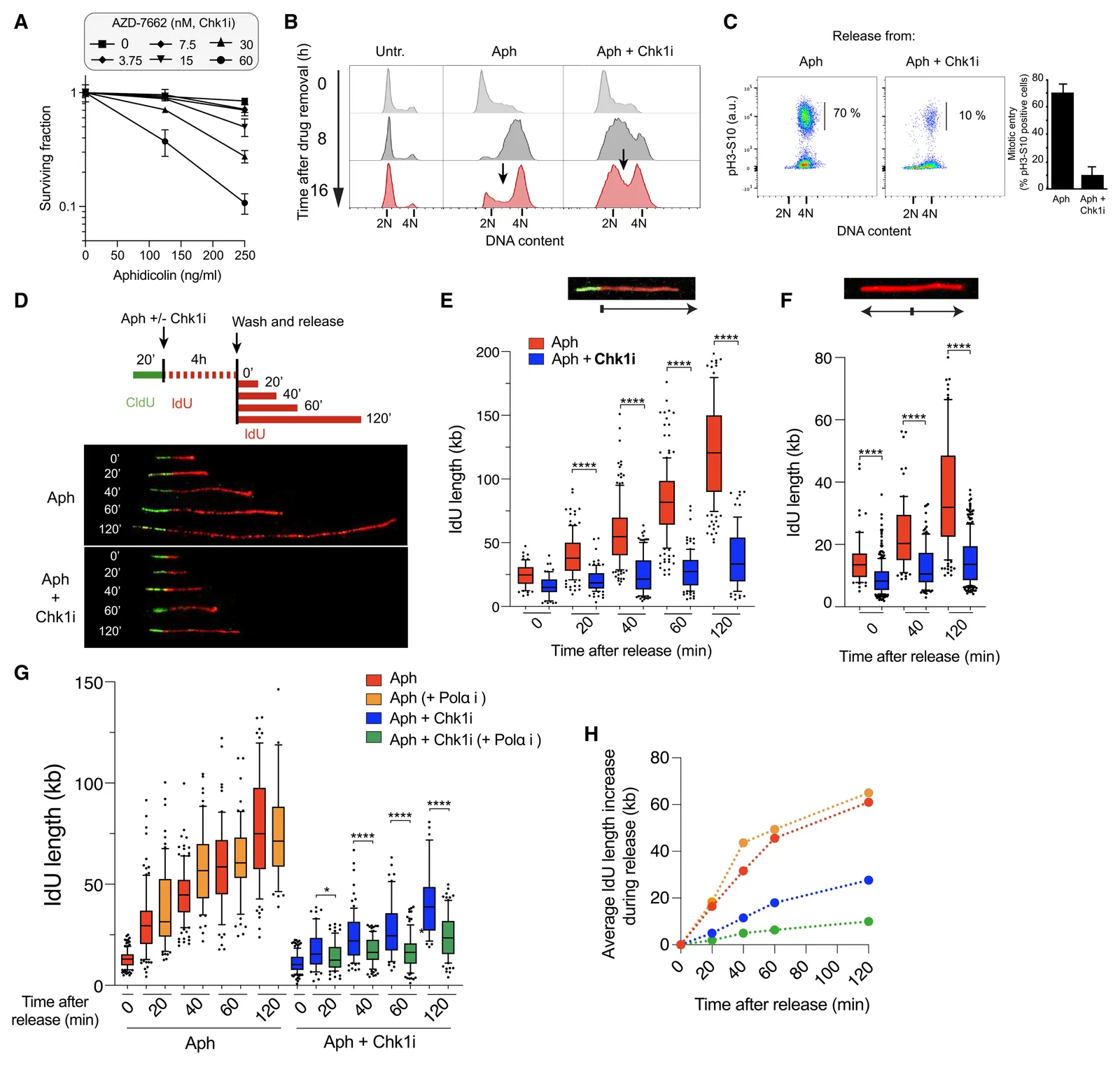
Checkpoint inhibition after replication fork stalling induces S-phase catastrophe (A) Survival assay of U2OS cells treated as in Figure S1A with indicated doses of Chk1i (AZD7762) in combination with APH. Viability was determined using CellTiter-Glo and normalized to APH-untreated cells. Error bars represent SEM. (B) S-phase progression analysis. Cells were treated as in Figure S1L and analyzed at 0, 8, and 16 h after release in media with Taxol. (C) Mitotic entry analysis. Cells were treated as in Figure S1L and analyzed at 16 h after release in media with Taxol. The proportion of pH3-S10-positive cells is shown. Error bars represent SEM. (D) DNA fiber analysis of individual replication fork progression. Upper panel, experimental setup: cells were pulsed with CldU for 20 min, washed, and incubated with IdU and APH alone or in combination with Chk1i for 4 h. Cells were then washed and released in media only with IdU for 20, 40, 60, and 120 min. Bottom panel, representative micrographs of CldU-IdU replication tracks. (E and F) Box-and-whisker plot and median IdU track length in bicolor fibers (forks fired before treatment, E) or red-only fibers (forks fired after treatment, F). (G) DNA fiber analysis of Polα-dependent synthesis following checkpoint inhibition. Experimental setup as in (D), but the Polα inhibitor ST1926 (4 μM) was added during release. Box-and-whisker plot and median IdU track length in bicolor fibers. Fiber data were analyzed using two-tailed Mann-Whitney U tests; *p < 0.05, ****p < 0.0001. (H) Average length increase for IdU tracks of bicolor fibers during release. See also Figure S1.

Consistent with previous work showing that loss of ATR or Chk1 causes a diverse array of pathological phenotypes,^1,11,17,37–41^ treatment of U2OS cells with ATR or Chk1 inhibitors (ATRi and Chk1i) caused cell lethality (Figures S1A and S1B), which was exacerbated by a variety of genotoxic agents (Figures 1A and S1C–S1F), including the DNA polymerase α, δ, and ε inhibitor aphidi-colin (APH). Addition of Chk1i during the last 4 h of a 24-h APH treatment increased DNA damage markers, chromatin-bound Rad51, and ssDNA derived from both parental and nascent DNA, consistent with previous work (Figures S1G–S1J).^38–40,42^ Checkpoint inhibition also led to RPA exhaustion,^37^ defined as chromatin association of large amounts of RPA, which, after reaching a threshold, is followed by a sharp increase in γH2AX levels (Figure S1K).^38–40,42^ Cells released from the APH/Chk1i treatment regime (Figure S1L) were largely unable to complete S phase and enter mitosis, in contrast to cells released from APH only, and the few cells that reached mitosis exhibited high levels of nuclear aberration (Figures 1B, 1C, and S1M). This suite of phenotypes, which includes, among others, unscheduled firing of replication origins, extensive stalling and collapse of replication forks, widespread DNA damage and breakage, and, ultimately, cell death, is termed S-phase catastrophe.^21,30,43^

Chk1 inhibition also had a profound effect on DNA replication fork restart (Figures 1D–1F), consistent with previous work.^38,40,44–46^ Cells were pulsed with CldU (green) for 20 min, CldU was washed out, and APH and IdU (red), with or without checkpoint inhibitors, were added. After 4 h, APH and IdU were washed out and IdU alone was added back (Figure 1D). Replication forks from cells released from APH-only treatment immediately began to synthesize DNA after APH removal at rates very similar to unperturbed forks (Figures 1D and 1E). Replication forks also resumed synthesis after APH/Chk1i treatment; however, fork rate was roughly 5 times slower (Figures 1D and 1E). As expected,^44,45,47–49^ transient checkpoint inhibition led to a significant increase in new origin firing, measured as tracks labeled only in red (Figure S1N). These red-only tracks were much shorter after Chk1i (Figure 1F), consistent with the idea that replication forks are affected by Chk1i regardless of whether they were established before or after checkpoint inhibitor addition. We did not detect nascent strand degradation in either APH- or APH/Chk1i-treated cells (Figure S1O) using a previously published approach^50^ (Figure S1P), indicating that the reduction in apparent extension rates observed after fork stalling in checkpoint-deficient cells is a consequence of reduced replication fork elongation rate, not increased degradation rate, consistent with previous work.^38,51^

Fork rate, when assessed by DNA fiber spreading, was not affected when the Polα inhibitor, ST-1926,^52,53^ was added during the restart of replication forks in APH-only cells; however, ST-1926 almost completely inhibited fork extension in APH/Chk1itreated cells (Figures 1G and 1H). Similar results were obtained with a second Polα inhibitor, CD-437^54^ (Figure S1Q). Although DNA fiber spreading cannot distinguish leading from lagging strands,^55^ the synthesis of many kilobases of DNA after APH treatment alone in the presence of the Polα inhibitor indicates efficient resumption of leading-strand replication in the absence of lagging-strand synthesis; the near complete inhibition of all synthesis after APH and Chk1i treatment in the presence of the Polα inhibitor can only be interpreted as loss of both leading- and lagging-strand synthesis. Therefore, the slow fiber extension during replication fork restart in checkpoint-deficient cells is dependent upon Polα, whereas the rapid fiber extension after release from APH-only does not require Polα and is more consistent with the resumption of processive leading-strand DNA synthesis by Polδ and/or Polε.

### Order of effects following checkpoint inhibition

**Figure 2:**
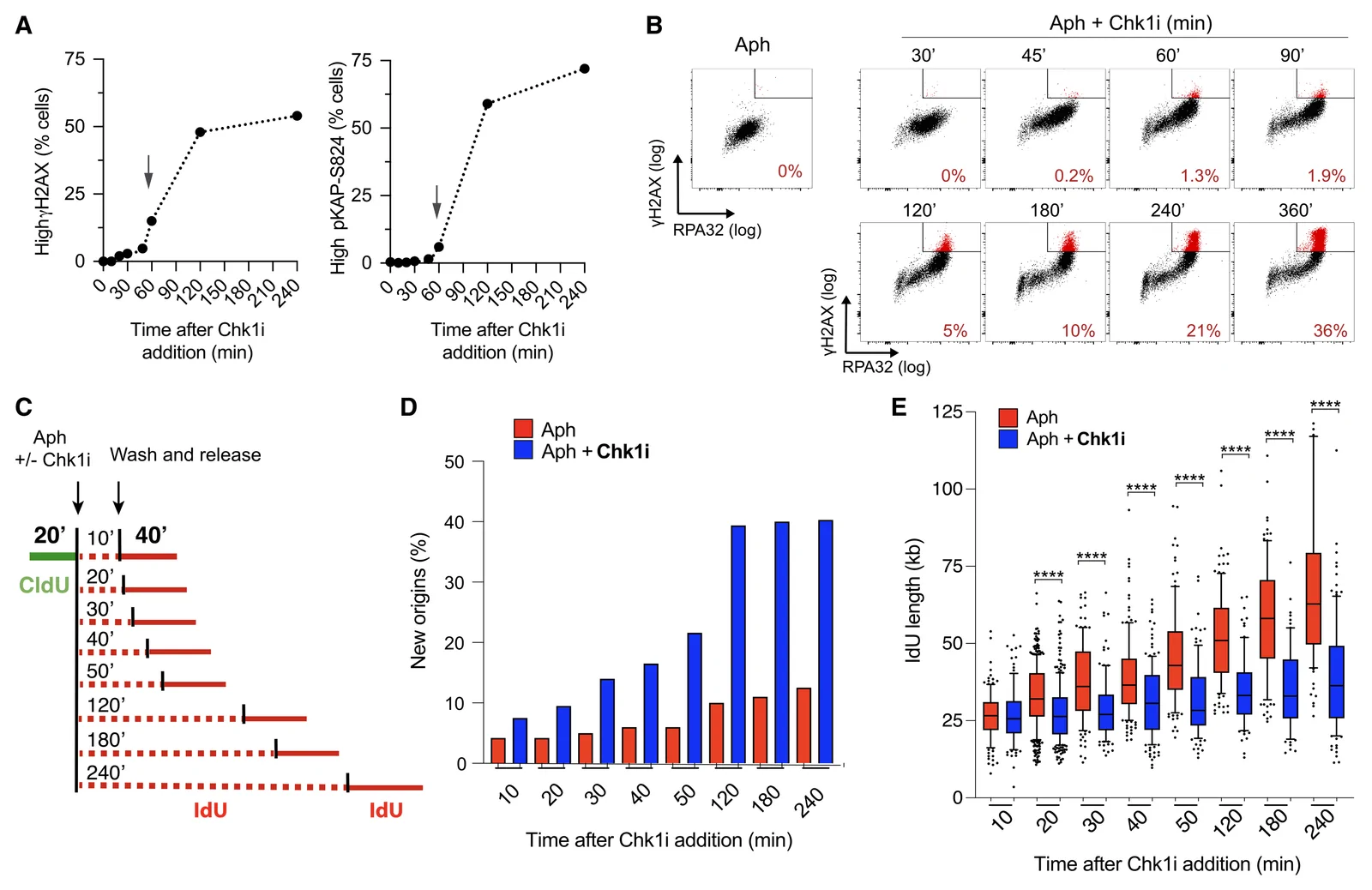
Order of effects following checkpoint inhibition (A and B) Flow cytometry analysis of DNA damage markers as depicted in Figure S1R. (A) Percentage of cells presenting high γH2AX and phospho-S824 KAP1 levels in cells treated with APH alone or in combination with Chk1i. (B) Levels of chromatin-bound RPA32 and γH2AX in the same population of cells. Representative images comparing γH2AX intensity (log scale) and chromatin-bound RPA32 intensity (log scale) in cells. Gated cells depicted in red represent cells with simultaneously high levels of γH2AX and RPA. Bottom right of each panel, percentage of red-colored cells. (C–E) DNA fiber analysis of replication fork progression and origin firing. (C) Experimental setup. Cells were pulsed with CldU for 20 min, washed, and then incubated with IdU plus 1 μg/mL APH alone or in combination with 50 nM Chk1i for the indicated time. Cells were then washed and released in media containing IdU for 40 min. (D) Percentage of new origins fired was determined by counting the number of IdU-only tracts over the total number of different replication structures. (E) Box-and-whisker plot and median IdU track length in bicolor fibers. Data were analyzed using two-tailed Mann-Whitney U tests; ****p < 0.0001. See also Figures S1R and S1S.

We next determined the order in which the myriad phenotypes first appeared after checkpoint inhibition. We exposed APH-treated cells to increasing durations of Chk1 inhibitor, from 10 min to 5 h (Figure S1R). DNA damage markers were almost undetectable until 45 min after checkpoint inhibitor addition (Figure 2A), whereas RPA exhaustion, defined as the point where γH2AX levels continue to rise without commensurate increase in chromatin-bound RPA, was first seen after 60 min of treatment and continued to accumulate over the next 4 h (Figure 2B). A clear increase in origin firing could be detected even at the earliest time points (10–20 min) after checkpoint inhibitor addition when compared with APH alone (Figures 2C, 2D, and S1S), consistent with previous work.^38^ Defective replication fork restart was also observed early, after 20 min of Chk1i treatment (Figure 2E). These results show that new origin firing and fork restart defects occur well before the onset of detectable DNA damage or depletion of chromatin-bound RPA, arguing that DNA damage generation or RPA exhaustion are likely to be downstream of new origin firing and/or fork restart defects following checkpoint inhibition.

### Excess origin firing depletes chromatin-bound PCNA and causes replication fork defects

**Figure 3:**
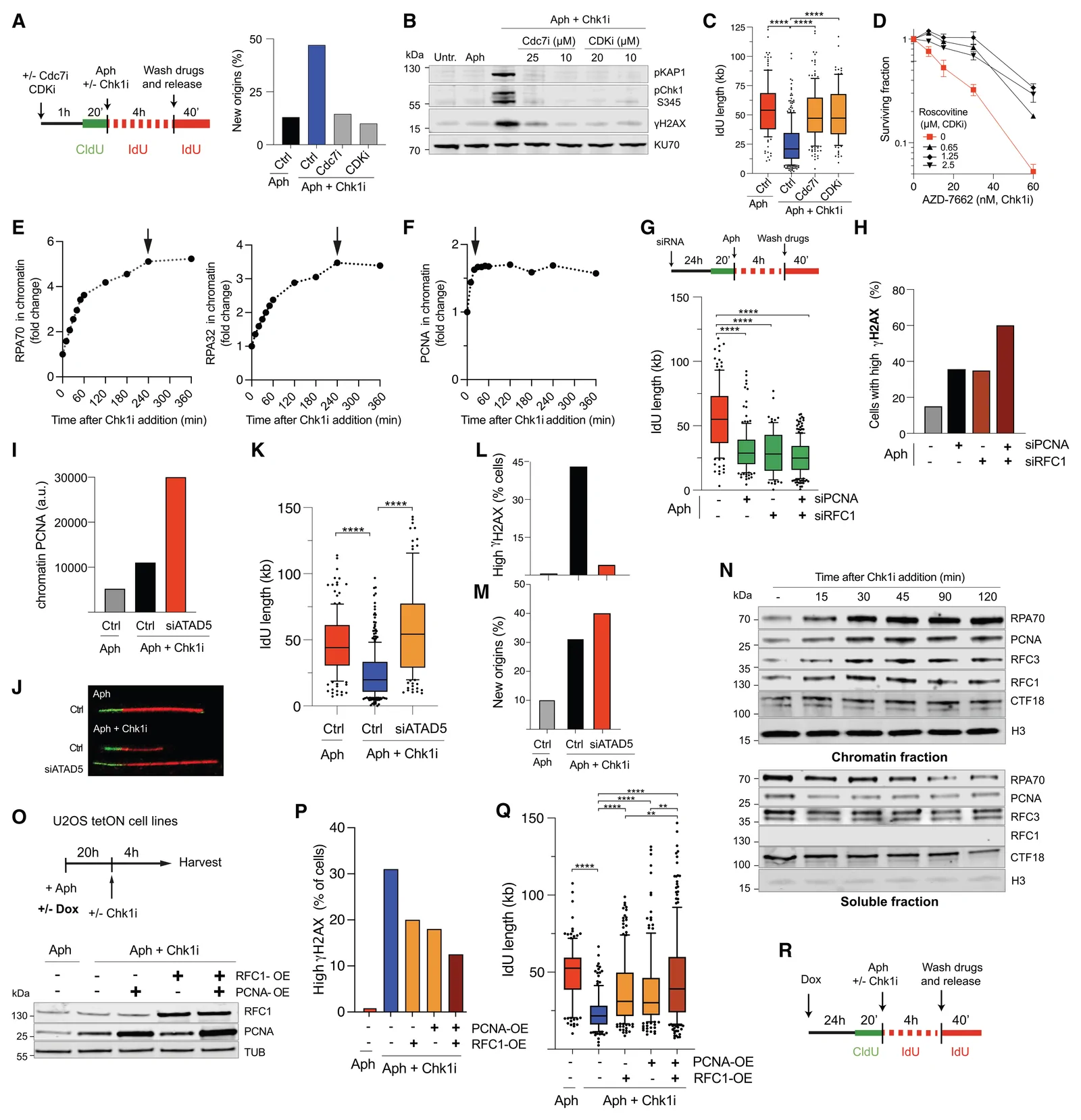
Excess origin firing depletes chromatin-bound PCNA and causes replication fork defects (A–C) Cells were treated with APH alone or in combination with Chk1i, with and without Cdc7i (XL-143) or CDKi (roscovitine). (A) Experimental setup (left panel). Percentage of new origins fired (right panel). (B) DNA damage marker immunoblots. (C) Box-and-whisker plot and median IdU track length in bicolor fibers. Cells were treated as shown in Figure 2A. (D) Survival assay of cells treated as in Figure S1A with indicated concentrations of Chk1i alone or in combination with roscovitine. Error bars represent SEM. (E and F) Chromatin-bound RPA32 and RPA70 (E) and PCNA (F) in APH/Chk1i-treated cells. Graphs show fold change of mean fluorescence intensities (MFIs) in comparison with APH alone. (G) Replication fork progression by DNA fiber analysis in APH-treated cells depleted for PCNA and RFC1 alone or in combination. Experimental setup (upper panel). Box-and-whisker plot and median IdU track length in bicolor fibers (bottom panel). (H) Percentage of cells presenting high levels of γH2AX in APH-treated cells depleted for PCNA and RFC1 alone or in combination. (I) MFI of chromatin-bound PCNA in ATAD5-depleted cells. (J) Representative micrographs of the experiment detailed in (K). (K) DNA fiber analysis of replication fork progression in ATAD5-depleted cells. Left, box-and-whisker plot and median IdU track length in bicolor fibers. (L) Percentage of control or ATAD5-depleted cells presenting high levels of γH2AX. (M) Percentage of new origins fired in control and ATAD5-depleted cells. (N) Immunoblot of chromatin extracts of APH-treated cells for 24 h alone or with the addition of 50 nM Chk1 inhibitor during the indicated times just before harvesting. Histone 3 (H3) was used as a loading control for chromatin fraction. (O) Immunoblots showing RFC1 and PCNA expression after 24 h doxycycline (Dox) induction in U2OS tetON-RFC1 and U2OS tetON-PCNA cells, respectively. Experimental setup (upper panel). (P) Flow cytometry analysis of the percentage of cells presenting high levels of γH2AX staining. (Q and R) Replication fork progression analysis in cells overexpressing PCNA, RFC1, or both. (Q) Box-and-whisker plots showing median IdU track length in bicolor fibers. (R) DNA fiber assay experimental setup. Fiber data were analyzed using two-tailed Mann-Whitney U tests; **p < 0.01,****p < 0.0001. See also Figure S2.

New origin firing and fork restart defects started at similar times after Chk1i treatment. Inhibition of excess origin firing using either CDC7 or CDK2 inhibitors suppressed DNA damage markers, RPA exhaustion, and the defect in replication fork restart associated with Chk1i (Figures 3A–3C and S2A), in agreement with previous work,^37,45^ and also rescued mitotic entry and viability (Figures S2B and 3D). This suggests that the generation of extra replication forks depletes some essential replication factor. It has previously been proposed that this limiting factor is RPA: treatment with hydroxyurea and checkpoint inhibitors generates sufficient ssDNA to exhaust the soluble pool of RPA, resulting in DNA damage, fork breakage, and, ultimately, fork collapse.^37^ However, our results indicate that fork defects precede RPA exhaustion. RPA overexpression (Figure S2C) rescued some of the DNA damage seen at 120 min and later (Figure S2D), consistent with previous work.^37^ However, it did not rescue the DNA damage observed at earlier time points. Moreover, RPA overexpression did not rescue the Chk1i-induced fork restart defects at any time point (Figures S2E and S2F). Consistent with this, chromatin-bound RPA levels are relatively low in the first 30 min when restart defects first occur but continue to rise steadily over time and do not plateau until 4 h of checkpoint inhibition (Figure 3E). Thus, RPA is unlikely to be the replication factor limiting for fork restart seen from early time points, though its depletion does contribute to DNA damage at later time points.

In sharp contrast to RPA, however, we found that the amount of PCNA bound to chromatin increased less than 2-fold and reached a maximum level within 30 min of checkpoint inhibition (Figure 3F) despite the fact that the number of new forks continued to rise over 2 h (Figures 2D and S1S). We therefore considered this decrease in the amount of PCNA per fork could be responsible for the replication fork restart defect, analogous to results in the accompanying manuscript.^56^ Consistent with this and with previous work,^57^ transient reduction of either PCNA or the PCNA clamp loader RFC1 (Figure S2G)affected replication restart (Figure 3G) and caused DNA damage (Figures 3H and S2H), even in the absence of excess origin firing and regardless of checkpoint status, suggesting that reduction of chromatin-bound PCNA levels prevents normal fork restart.

Depletion of ATAD5/Elg1,^58,59^ the main unloader of PCNA from chromatin, led to a marked increase in chromatin-bound PCNA (Figure 3I), which almost completely suppressed the Chk1i-induced fork restart defects and accumulation of DNA damage markers (Figures 3J–3L), although having no effect on replication or DNA damage in APH-treated cells (Figures S2I and S2J). Codepletion of PCNA, but not Rad18, with ATAD5 prevented suppression of DNA damage (Figures S2K and S2L), arguing that suppression is due to the increased chromatin-bound PCNA rather than K164 mono-ubiquitylation of PCNA. Importantly, ATAD5 depletion did not prevent excess origin firing (Figure 3M), consistent with previous work.^60^ We re-examined published high-throughput CRISPR screening datasets and found that ATAD5 was among the top 10 hits in a whole-genome CRISPR-Cas9 dropout screen in RPE1 cells for genes whose loss conferred resistance to the ATR inhibitor AZD6738,^61^ ranking higher than known sensitivity determinants such as Cdc25A and Cdc25B.^62^ ATAD5 also emerged as a comparably strong hit in a similar whole-genome CRISPR-Cas9 dropout screen for AZD6738 in mouse embryonic stem cells.^63^

Transient overexpression of PCNA in a doxycycline-inducible U2OS cell line (Figure 3O, lane 3) led to a significant reduction in DNA damage markers and an improvement in replication fork restart (Figures 3P–3R). PCNA is an abundant protein, and, consistent with this, even after 120 min of Chk1inhibitor exposure, PCNA, as well as RPA, were detected in soluble as well as chromatin fractions (Figure 3N). Similarly, RFC3, one of the core RFC subunits present in all RFC complexes, was also detected in both fractions. By contrast, RFC1, which is roughly 10-fold less abundant than the core RFC subunits and ~ 20-fold less abundant than PCNA,^64^ was detectable on chromatin but not in the soluble fraction, even at the earliest times (Figure 3N). Consistent with this, transient overexpression of RFC1 (Figure 3O, lane 4) also reduced DNA damage accumulation and replication fork progression defects (Figures 3P–3R). Co-overexpression of PCNA and RFC1 (Figure 3O, lane 5) resulted in a more pronounced rescue of both phenotypes (Figures 3P–3R), supporting the idea that both chromatin-bound PCNA and RFC1 are limiting under these conditions.

### HLTF is responsible for the defects caused by RFC/PCNA-depleted forks in the absence of the checkpoint

Our results thus far show that excess origin firing drives RFC/PCNA depletion, which prevents normal replication restart. They do not, however, explain where the DNA damage and excessive ssDNA come from or why the fork arrest is irreversible. We developed an RNA interference-based genome-wide screen in human cells transiently treated with APH and Chk1 inhibitors, where we monitored γH2AX levels as a marker for DNA damage and the mitotic phosphorylation of H3 (pH3-S10) as an indicator of S/G2 phase completion (for further details see Figures S3 and Tables S1 and S2). When focusing on the small interfering RNAs (siRNAs) that increased γH2AX levels, we found that out of the ~21,000 genes tested, RFC2 depletion emerged as the highest-scoring gene, RFC1 depletion ranked among the top 20, and all RFC subunits as well as PCNA increased γH2AX levels (Figure S3E), further supporting the idea that RFC1–5 is the limiting factor preventing normal fork restart.

**Figure 4:**
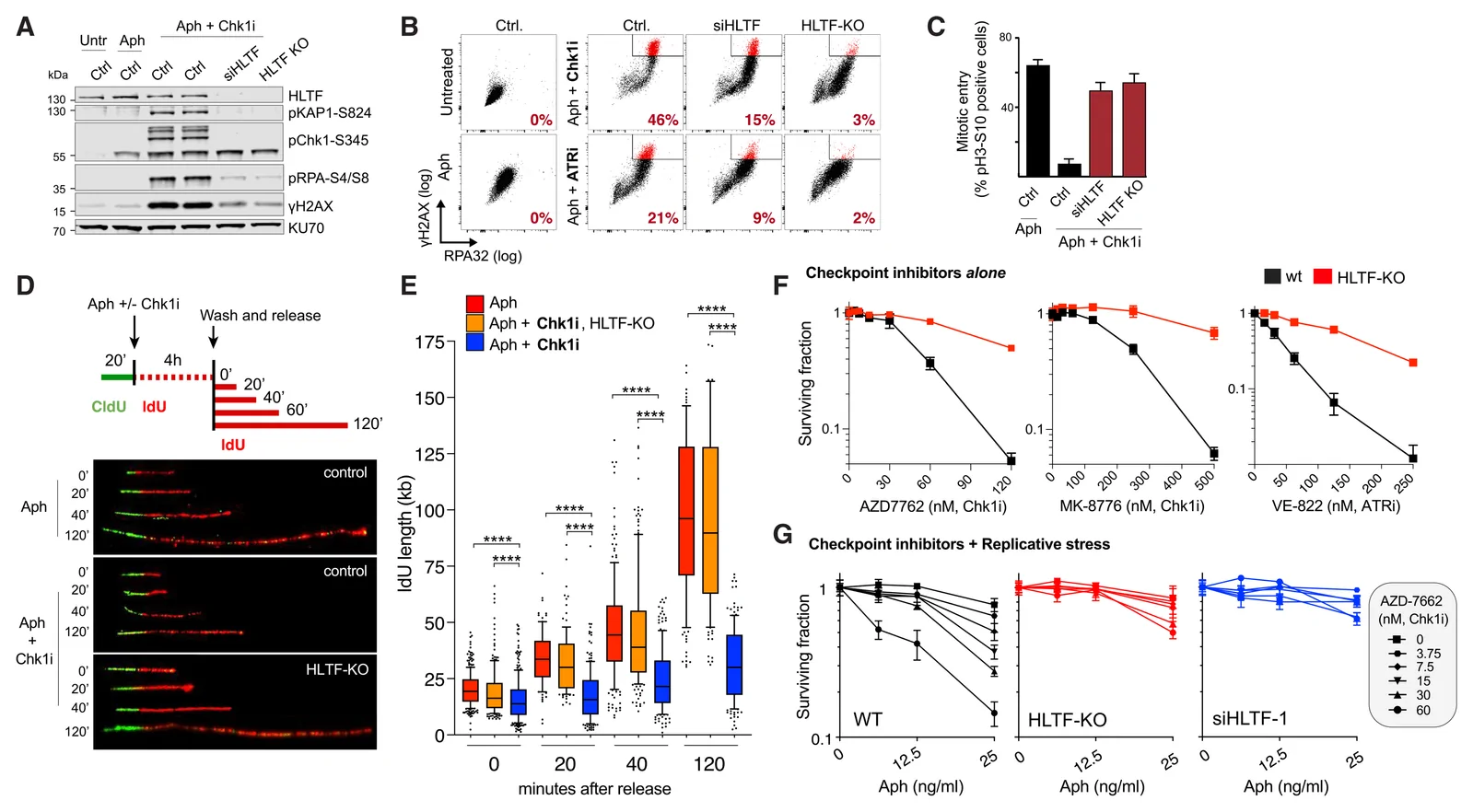
HLTF is responsible for the defects caused by RFC/PCNA-depleted forks in the absence of the checkpoint (A) Immunoblots for DNA damage markers in control, HLTF siRNA knockdown (siHLTF), and HLTF CRISPR knockout (KO) cells after checkpoint inhibition. (B) Flow cytometry analysis of chromatin-bound RPA32 and γH2AX levels in the same population of cells. Gated cells depicted in red represent cells with simultaneously high levels of γH2AX and RPA. Bottom right of each panel, percentage of red-colored cells. (C) Mitotic entry analysis. Cells were treated with APH for 24 h, with or without Chk1i during the final 4 h. Cells were washed and released for 16 h. The proportion of pH3-S10-positive cells is shown. Error bars represent SEM. (D) (D and E) Kinetic analysis of replication fork progression and origin firing in checkpoint-inhibited conditions by DNA fiber. (E) Upper panel, experimental setup. Bottom panel, representative micrographs. (F) Box-and-whisker plot and median IdU track length in bicolor fibers. Data were analyzed using two-tailed Mann-Whitney U tests; ****p < 0.0001. (F and G) Survival assay of HLTF-KO or KD cells treated with indicated concentrations of Chk1i (AZD7762 and MK-8776) or ATRi (VE-822) alone (F) or in combination with APH (G). Error bars represent SEM. See also Figures S3–S6.

Next, we identified genes whose knockdown reduced DNA damage and promoted mitotic entry after Chk1i removal, indicating S-phase completion through fork resumption. After secondary screens and siRNA deconvolution, one of the strongest such suppressors was HLTF (also known as SMARCA3) (Figure S3F; Table S2). HLTF is a fork-associated protein, ubiquitin ligase, and SNF2-family DNA translocase implicated in several DNA repair/tolerance pathways.^65–70^ Moreover, HLTF inactivation reduces DNA damage and slightly increases resistance to genotoxic agents and ATR inhibitors.^66^ We found that both HLTF siRNA depletion and CRISPR-Cas9 gene knockout (HLTF-KO) strongly reduced phosphorylation of all DDR markers analyzed in APH-treated cells after Chk1 or ATR inhibition (Figures 4A and S4A). Complementation of HLTF-KO cells with full-length HLTF cDNA (Figure S4B) restored the elevated DNA damage (Figure S4C) and replication fork defects (Figure S4D) induced by checkpoint inhibition. RPA exhaustion, high levels of parental and nascent ssDNA, Rad51 foci, and strand breaks were almost completely suppressed in APH/Chk1i cells in the absence of HLTF (Figures 4B and S4E–S4G). This reduction in S-phase-related DNA damage in HLTF-deficient cells was accompanied by enhanced levels of mitotic entry and a strong reduction in Chk1i-induced genomic instability (Figures 4C and S4H), consistent with these cells being able to resume replication and complete S phase (Figure S4I). HLTF depletion and gene KO suppressed the replication restart defects in ATR- or Chk1-inhibited cells from replication forks established either before or after Chk1 inhibition (Figures 4D, 4E, S4J, and S4K). Replication forks initiated either before or after checkpoint inhibition show similarly impaired fork progression (Figures 1E and 1F) and are equally rescued by HLTF inactivation (Figures 4E and S4K), indicating that newly fired origins are just as susceptible to collapse, and just as responsive to HLTF loss, as pre-existing forks.

The suppression of restart defects seen in HLTF-deficient cells occurred despite the excess origin firing and increase of chromatin-bound PCNA (Figures S4L–S4N). We re-examined publicly available high-throughput CRISPR screening datasets and noted that HLTF ranked among the top 5 hits in CRISPR dropout screens for resistance to ATR and Chk1 inhibitors using a DNA damage sgRNA sub-library.^71^ Moreover, it scored among the top hits in whole-genome CRISPR-Cas9 screens in MCF10^72^ and RPE1^61^ cells for resistance to the ATR inhibitor AZD6738.

To further investigate the drivers of fork collapse, we examined cellular processes involving HLTF. HLTF, along with the E2 ubiquitin-conjugating complex MMS2/UBC13, polyubiquitylates PCNA in response to DNA damage to initiate error-free lesion by-pass via template switching.^68,73,74^ We generated MMS2 and UBC13 CRISPR-KO cells, neither of which suppressed DNA damage in the absence of checkpoint activity (Figure S5A), suggesting that PCNA polyubiquitination is not involved in fork collapse. In addition, an HLTF mutant (C760S) inactivating the RING domain, introduced via CRISPR-Cas9 in eHAP cells, also failed to suppress checkpoint-inhibition-induced DNA damage (Figure S5B) and lethality (Figure S5C), further supporting this conclusion.

SHPRH, like HLTF, is a human Rad5 ortholog with a RING domain and SWI/SNF-ATPase helicase but lacks the HIRAN domain.^69,74,75^ We generated SHPRH KO cells, and they show replication fork defects and levels of DNA damage marker phosphorylation similar to controls after checkpoint inhibition, suggesting no involvement in this process (Figures S5D–S5G). Genetic and biochemical studies suggest that replication fork reversal^76^ is predominantly catalyzed by the SNF2-family DNA translocases HLTF,^65,66^ SMARCAL1,^77^ and ZRANB3,^78,79^ and SMARCAL1 has been shown to be required for the generation of nascent-strand ssDNA in human cells when ATR is inactivated.^38^ So, we tested whether SMARCAL1 or ZRANB3 are required to promote fork collapse in the absence of a functional checkpoint. Individual and double CRISPR-KO U2OS cell lines of SMARCAL1 and ZRANB3 did not suppress Chk1-inhibitioninduced replication fork defects, phosphorylation of DNA damage markers, generation of parental ssDNA, or accumulation of RPA (Figures S5H–S5L). Moreover, epistasis experiments revealed that HLTF depletion suppressed Chk1-inhibition-induced DNA damage markers, regardless of ZRANB3 or SMARCAL1 status (Figure S5M). These findings suggest that checkpoint-inhibition-induced fork collapse does not require HLTF’s PCNA polyubiquitination activity, SHPRH, or the fork remodelers SMARCAL1 and ZRANB3 but instead points to a unique role for HLTF in this process.

**Figure 5:**
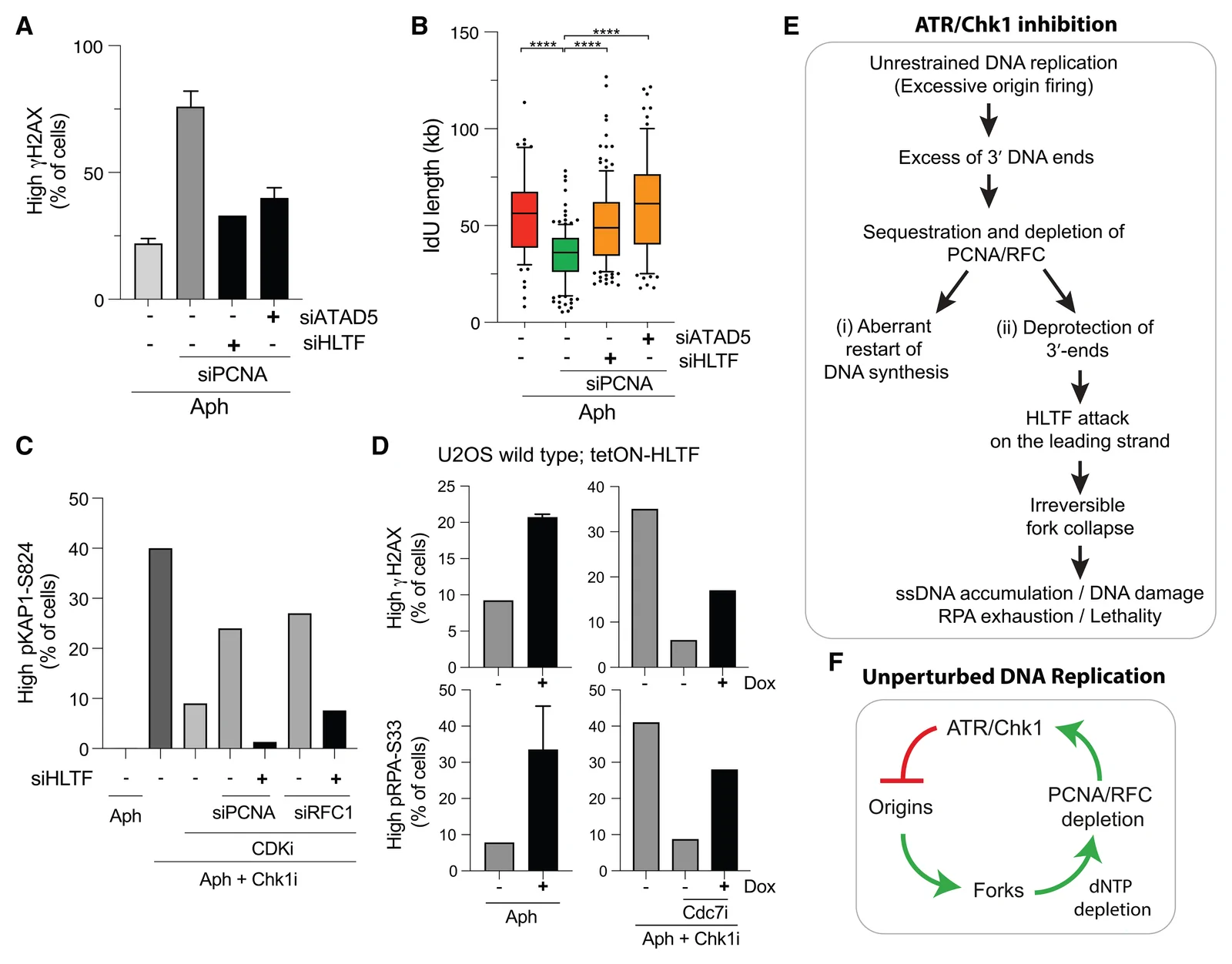
HLTF is responsible for the defects caused by RFC/PCNA-depleted forks (A) Flow cytometry analysis of the percentage of APH-treated cells presenting high levels of γH2AX chromatin staining in control cells and PCNA siRNA-treated cells alone or in combination with HLTF or ATAD5 siRNA. (B) DNA fiber analysis of replication fork progression in APH conditions in control cells and PCNA siRNA-treated cells alone or in combination with HLTF or ATAD5 siRNA. Cells were pulsed with CldU for 20 min, washed, and then incubated with IdU plus 1 μg/mL APH for 4 h. Cells were then washed and incubated again only with IdU for 40 min. Box-and-whisker plot and median IdU track length in bicolor fibers. At least 120 forks were scored per sample. Data were analyzed using two-tailed Mann-Whitney U tests; ****p < 0.0001. (C) Flow cytometry analysis of the percentage of cells presenting high levels of pKAP1 staining. Control cells were treated with 1 μg/mL APH for 24 h alone. Control cells and cells treated with PCNA or RFC1 siRNA alone or in combination with HLTF siRNA were subjected to APH for 24 h and a checkpoint inhibitor, with or without a CDKi (roscovitine, 5 μM), was added during the final 4 h. (D) Flow cytometry analysis of the percentage of tetON-HLTF wild-type U2OS cells presenting high levels of DNA damage markers (γH2AX and pRPA-S33) after treatment with APH alone or in combination with Chk1i, with or without Cdc7i (XL-143, 10 μM). Flow cytometry data; error bars represent SEM. (E) Proposed order of events and dependencies during replication fork collapse in the absence of a functional checkpoint. (F) Model for coordination of fork progression and origin firing via checkpoint activation and dNTP/RFC/PCNA depletion.

Our results suggest that depletion of PCNA/RFC by excess OkF synthesis from excess origins generates a substrate for HLTF to act upon, leading to fork collapse. In line with this, ATAD5 and HLTF depletion alleviated the effects observed when PCNA and/or RFC1 are depleted without excess origin firing and regardless of checkpoint status (Figures 5A–5C). This suggests that PCNA retention on chromatin can protect free 3' ends from nucle-ase attack and that HLTF can cause fork restart defects even in the presence of a functional checkpoint. Consistent with this, overexpressing HLTF without fork stalling or checkpoint inhibition led to an increase in DNA damage markers (Figure 5D). These results suggest that the checkpoint may protect replication forks from HLTF even in the absence of genotoxic stress.

HLTF-deficient cells showed increased resistance to ATR and Chk1 inhibitors, both in the absence (Figures 4F and S6G) and in the presence (Figures 4G, S6A–S6F, and S6H–S6J) of a variety of genotoxic agents, including hydroxyurea, cisplatin, and the PARP inhibitor olaparib. Although APH was used as the primary source of replication stress throughout this study, these findings indicate that our conclusions are not restricted to DNA polymerase inhibition.

To test the generalizability of our model, we depleted HLTF and ATAD5 in multiple cell types, including cancer (HeLa, eHAP), immortalized (RPE-1), and transformed (HEK-293) lines, and consistently observed rescue of DNA damage, fork progression, and viability defects after checkpoint inhibition (Figures S5B and S6K–S6V).

## Discussion

The work described here, together with an accompanying manuscript^56^ showing the detailed molecular mechanism through biochemical reconstitution of DNA replication fork stalling and restart, provides a framework for understanding how the DNA replication checkpoint stabilizes stalled replication forks in human cells (Figure 5E). When DNA replication origin firing is not restrained by the checkpoint after inhibition of DNA synthesis, excess OkFs are initiated; these OkFs sequester PCNA and the canonical RFC1-containing RFC complex, which becomes specifically depleted. As incomplete OkFs accumulate (OkF synthesis cannot complete because of DNA polymerase inhibition, see accompanying manuscript^56^), the depletion of PCNA/RFC exposes 3' DNA ends and it is the action of HLTF on these exposed ends that causes fork collapse (Figure 5E). HLTF localizes to replication forks even in the absence of genotoxic stress^65^ and promotes fork reversal *in vivo* and *in vitro* via a HIRAN domain that binds to 3' DNA ends.^65,66,80,81^ We propose that the irreversible effect of HLTF on replication forks is the initiation of unwinding of the nascent leading strand,^82^ which exposes ssDNA from both the nascent and parental strands (Figure S4F) and depletes RPA (Figure 4B), consistent with previous work.^37,38,40,42^ By eliminating the nascent leading strand, any continued unwinding by CMG after restart can only support lagging-strand synthesis, which explains why all synthesis after APH/Chk1i treatment is slow and is sensitive to Polα inhibitors (Figures 1G, 1H, and S1Q). Although PRIMPOL may be able to generate primers on the leading-strand template,^83^ these cannot be efficiently extended under conditions of PCNA/RFC depletion.

Our findings suggest a parallel with the established function of the fork protection complex (comprising proteins such as BRCA1, BRCA2, and FANCD2), which prevents nucleolytic degradation of reversed replication forks by enzymes like MRE11 and DNA2.^50,84,85^ In a conceptually analogous manner, PCNA and RFC1 engagement at stalled, but not reversed, forks could similarly protect exposed 3' DNA ends, limiting access by enzymes such as HLTF, thereby preventing fork collapse.

This work, together with the accompanying manuscript,^56^ indicates that the primary function of the DNA replication checkpoint in stabilizing stalled replication forks is to prevent the accumulation of excess OkFs by inhibiting CMG unwinding and/or inhibiting new origin firing. The work in this paper emphasizes the importance of regulating new origin firing in human cells, whereas the accompanying paper^56^ emphasizes the importance of regulating CMG unwinding via Mrc1 and Mcm10 phosphorylation. We do not currently know whether human MCM10 or Claspin (the human ortholog of Mrc1) are regulated by Chk1. Similarly, we know that late origin firing is also regulated by the checkpoint in yeast, but its relative importance in restricting OkF accumulation is unknown. Finally, HLTF and its yeast ortholog Rad5 have distinct biological roles. HLTF promotes fork reversal,^65,66^ and its loss suppresses fork defects, DNA damage, and lethality when checkpoint activity is compromised. In contrast, Rad5 is a master regulator of DNA damage tolerance in yeast, and its deletion sensitizes cells to genotoxic stress.^86^ Whether Rad5 also plays a role in fork collapse in yeast is currently unknown.

Our work also provides insights into the role of the checkpoint in coordinating replication during an unperturbed S phase. HLTF promotes cell lethality after checkpoint inhibition even *without* added replicative stress (Figure 4F), HLTF is responsible for the DNA damage induced by partial PCNA or RFC knockdown even in the presence of a functional checkpoint (Figures 5A–5C), and HLTF overexpression causes DNA damage even in the presence of a functional checkpoint (Figure 5D). These results suggest that the balance between HLTF, RFC/PCNA/3' ends, and the DNA replication checkpoint is crucial, even during an unperturbed S phase (Figure 5F). We suggest that, upon entry into S phase, origin activation continues until the number of forks begins to deplete RFC. RFC depletion causes uncoupling of leading-strand synthesis from CMG progression and prevents OkF completion and maturation, generating ssDNA on the leading-and lagging-strand templates that drive checkpoint activation. Excess origin activation also causes dNTP depletion,^41^ which, as shown in the accompanying manuscript,^56^ can promote RFC/PCNA depletion and thus may also contribute to checkpoint activation. Checkpoint activation then inhibits new origin firing, preventing further RFC/PCNA depletion; as OkFs mature and replicons terminate, RFC and PCNA are released and recycled and, as RFC depletion is relieved, the checkpoint signal is quenched, allowing new origins to fire. Thus, RFC/PCNA/3' end homeostasis, reinforced by dNTP depletion, ensures a constant level of origin firing throughout S phase (Figure 5F). Consistent with this model, Chk1 is active throughout unperturbed S phase,^87^ and inhibition of the checkpoint during unperturbed S phase leads rapidly to increased origin firing,^38,44,45,49,88^ indicating that the checkpoint is constantly suppressing new origin firing. Conditions that limit origin activation should prevent RFC depletion and accelerate fork rate, and, consistent with this, previous work has shown that reducing origin firing by manipulating the levels of CDC7, CDK, or ORC1 results in faster unperturbed fork rates, supporting this view.^89,90^

HLTF is frequently silenced by promoter hypermethylation in colon, gastric, esophageal, and other cancers,^80^ and HLTF KO promotes malignant transformation in mice.^91^ Additionally, tumor cells express high levels of PCNA^92^ and RFC,^93,94^ presumably to accommodate their high degree of uncontrolled replication and to overcome PCNA/RFC depletion. This is also consistent with *Atad5* heterozygous mice being cancer prone^95,96^ and with *Atad5* being identified as a genetic risk locus in genome-wide association studies for breast cancer^97^ and serous ovarian cancer.^98^ This suggests that HLTF functions as a tumor suppressor and that maintaining RFC/PCNA/3' end homeostasis may serve as a tumor-suppressive mechanism. We hypothesize that loss of HLTF may make some cancer cells—perhaps those with compromised DNA checkpoints or with dysregulated origin firing—resistant to chemotherapies. This may be especially important as a route for the emergence of drug resistance in cancers treated with DNA checkpoint inhibitors. Defining the balance and functional interplay between factors promoting fork collapse, like HLTF and ATAD5, and protective factors, like PCNA and RFC1, at replication forks could establish a predictive framework for determining cellular sensitivity to checkpoint inhibitors.

### Limitations of the study

Our experiments were primarily conducted in U2OS cells, a human osteosarcoma cell line. Although we extended our analysis to additional models, including HeLa, RPE1, HEK-293, and eHAP cells, these systems may not fully capture the complexity of *in vivo* cellular contexts or tumor heterogeneity. As such, further validation in animal models and patient-derived samples will be essential to assess the broader applicability and clinical relevance of our findings.

Our analysis focused on specific markers of DNA damage and replication stress, such as γH2AX and pKAP1-S824, and replication fork progression assays. Although these are well established indicators, they provide a limited view of the complex cellular responses to replication stress and checkpoint inactivation.

Lastly, the study did not assess the long-term consequences of dysregulating the proposed model on mutagenesis, genome stability, or tumorigenesis—areas that warrant further investigation.

## Resource Availability

### Lead contact

Further information and requests for resources and reagents should be directed to, and will be fulfilled by, the lead contact, John F.X. Diffley (john.diffley@ crick.ac.uk).

### Materials availability

- All unique strains and plasmids generated in this study will be made available upon request to the lead contact but may require a completed Materials Transfer Agreement.
- This study did not generate new unique reagents.

## Star★Methods

### Key Resources Table

**Table:** 

REAGENT or RESOURCE	SOURCE	IDENTIFIER
Antibodies		
Rabbit monoclonal anti-HLTF	Abcam	Cat#ab183042;RRID: AB_3095312
Mouse monoclonal anti-alpha-Tubulin	Sigma-Aldrich	Cat#T9026; RRID: AB_477593
Mouse monoclonal anti-BrdU	BD Biosciences	Cat#347580;RRID: AB_400326
Rat monoclonal anti-BrdU	Abcam	Cat#ab6326; RRID: AB_305426
Goat anti-Rabbit IgG (H+L)cross-adsorbed polyclonal secondary antibody, AlexaFluor 647 conjugated	ThermoFisher Scientific	Cat#A11006; RRID: AB_2534074
Goat anti-Rabbit IgG (H+L) cross-adsorbed polyclonal secondary antibody, AlexaFluor 594 conjugated	ThermoFisher Scientific	Cat#A-11012;RRID: AB_2534079
Mouse anti-SMARCAL1	Santa Cruz	Cat#:sc-376377; RRID: AB_10987841
monoclonal mouse anti-g-H2AX clone JBW301	Millipore	Cat#05-636; RRID: AB_309864
Monoclonal mouse anti-PCNA	Santa Cruz	Cat#sc56; RRID: AB_628110
Goat Polyclonal Alexa Fluor 488 anti rabbit IgG	Thermo Fisher	Cat#A11034; RRID: AB_2576217
IRDye 800CW Donkey anti-Rabbit IgG (H + L	LI-COR bioscience	Cat#926-32213
IRDye 680LT Donkey anti-Mouse IgG (H + L)	LI-COR bioscience	Cat#926-68022
Phospho KAP-1 (S824) anti-rabbit Antibody	Bethyl Laboratories	Cat#A300-767A
Phospho-RPA32 (Ser4, Ser8) Polyclonal Antibody	Bethyl Laboratories	Cat#A300-245A
Anti-Chk1 (phospho S345) (133D3) antibody anti rabbit	Cell Signaling	Cat#2348
Ku-70 antibody (A-9) anti-mouse	Santa Cruz	Cat#sc-5309
RFC3 anti-rabbbit	Abcam	Cat#ab182143
Polyclonal rabbit anti-ubiquityl PCNA(Lys164)	Cell Signaling	Cat#2577; RRID: AB_2118010
RPA32, 9H8, anti-mouse	Abcam	Cat#ab2175
RPA70 anti-rabbit	Abcam	Cat#EPR3472
RPA (Ab-2) Mouse mAb	Calbiochem	Cat#NA18
Rad51 Antibody (H-92) anti-rabbit	Santa Cruz	Cat#sc-8349
Histone 3 (H3) anti-mouse	Abcam	Cat#ab1791
pHistone 3-S10 anti-rabbit	Abcam	Cat#ab14955
pChk1(S296) antibody anti-rabbit [EPR915]	Abcam	Cat#ab79758
MMS2 polyclonal antibody anti-rabbit	Abcam	Cat#ab155007
UBC13 Monoclonal Antibody (4E 11)	Thermo Fisher	Cat#37-1100
SHPRH polyclonal antibody anti-rabbit	Abcam	Cat#ab80129
ZRANB3 Polyclonal Antibody anti-rabbit	Proteintech	Cat#23111-1-AP
CTF18 Antibody (F-1) anti-mouse	Santa Cruz	Cat#sc-374632
Bacterial and virus strains		
E.coli:DH5a	NewEnglandBioLabs	Cat#C2987
Chemicals, peptides, and recombinant proteins		
DAPI (4’,6-Diamidino-2-phenylindoledihydrochloride)	Sigma-Aldrich	Cat#32670
CldU (5-Chloro-2’-deoxyuridine)	Sigma-Aldrich	Cat#C6891
IdU (5-Iodo-2’-deoxyuridine)	Sigma-Aldrich	Cat#I7125
cOmplete Protease Inhibitor Cocktail, EDTA-Free, Mini Tablets	Sigma-Aldrich	Cat#11836170001
DMSO (Dimethyl Sulfoxide)	Millipore	Cat#MX1458-6
Puromycin	Invivogen	Cat#ant-pr-1
Hydroxyurea (HU)	Sigma-Aldrich	Cat#H8627
RNaseA	Sigma-Aldrich	Cat#R5503
Lipofectamine RNAi MAX	ThermoFisher	Cat#13778100
Fetal bovine serum, Tet Free	Sigma-Aldrich	Cat#F2442
Opti-MEM Reduced Serum Medium,GlutaMAX Supplement	ThermoFisher	Cat#51985034
Lipofectamine 3000	ThermoFisher	Cat#L3000008
Blasticidin	Invivogen	Cat#ant-zn-1
Zeocin	Invivogen	Cat#ant-bl-05
AZD7762, Chk1 inhibitor	AxonMEDCHEM	Cat#1399
VE-822, ATR inhibitor	Selleckchem	Cat#S7102
AlexaFluor 488 NHS Ester	ThermoFisher	Cat#A20000
MK-8776, Chk1 inhibitor	Selleck	Cat#S2735
VE-821, ATR inhibitor	Selleck	Cat#S8007
AZD6738, ATR inhibitor	Selleck	Cat#S7693
Doxycycline	Sigma-Aldrich	Cat#D9891
Pro-Long Gold Anti-fade Mountant	Invitrogen	Cat#P36930
Olaparib	Selleck	Cat#S1060
Cisplatin	Sigma-Aldrich	Cat#BP809
BrdU(5-Bromo-20-deoxyuridine)	Sigma-Aldrich	Cat#B5002
Ampicillin sodium salt	Sigma-Aldrich	Cat#A0166
Cristal Violet	Sigma-Aldrich	Cat#C6158-50G
Dulbecco’s Modified Eagle’s	Gibco	Cat#41966052
Medium(DMEM)- high glucose		
Taxol	Sigma-Aldrich	Cat#PHL89806-10MG
Cytochalasin-B	Sigma-Aldrich	Cat#C6762-1MG
BMS-863233 (XL-413), Cdc7 inhibitor	Axon	Cat#S7547-SEL
CD437, DNA-polymerase α inhibitor	Sigma-Aldrich	Cat#C5865-5MG
ST1926, DNA-polymerase α inhibitor	MedChem	Cat#HY-14808
Roscovitine	Sigma-Aldrich	Cat#R7772
Critical commercial assays		
QIAquick Gel Extract kit	QIAGEN	Cat#28706
QIAprep Spin Miniprep Kit	QIAGEN	Cat#27106
QIAGEN Plasmid MidiKit	QIAGEN	Cat#12143
CometAssay Electrophoresis Starter Kit	Trevigen (Bio-Techne)	Cat#4250-050-ESK
Deposited data		
Original microscopy and Western blots	This paper	Mendeley data:https://doi.org/10.17632/9k5cnmrvxk.1
Experimental models: Cell lines		
Human: U2OS	ATCC	Cat#HTB-96
Human: hTERT-RPE1	ATCC	Cat#CRL-4000
Human: HEK293T	ATCC	Cat#CRL-3216
Human: HeLa	ATCC	Cat#CRM-CCL-2
Human: Fully-haploid engineered HAP1 (eHAP) cells	Horizon	Cat#C669
Human: U2OS-HLTF KO	This paper	N/A
Human: U2OS TetON-PCNA	This paper	N/A
Human: U2OS TetON-RFC1	This paper	N/A
Human: U2OS TetON-PCNA, RFC1	This paper	N/A
Human: U2OS ZRANB3 KO	This paper	N/A
Human: U2OS ZRANB3 KO	This paper	N/A
Human: U2OS ZRANB3; SMARCAL1 KO	This paper	N/A
Human: U2OS MMS2 KO	This paper	N/A
Human: U2OS UBC13 KO	This paper	N/A
Human: U2OS SHPRH KO	This paper	N/A
Human: eHAP HLTF KO	This paper	N/A
Human: eHAP-RING C760S KI	This paper	N/A
Human: U2OS SuperRPA	Gift from Laboratory of Jiri Lukas, University of Copenhagen	N/A
Human: U2OS-HLTF KO; tetON-HLTF wild type	This paper	N/A
Oligonucleotides		
SMARTpool On TARGETplus Human ATAD5 siRNA	Dharmacon	Cat#L-004738-00
SMARTpool ON-TARGETplus Human HLTF siRNA-1	Dharmacon	Cat#L-006448-00
SMARTpool ON-TARGETplus Human PCNA siRNA	Dharmacon	Cat#L-003289-00
SMARTpool ON-TARGETplus Human RFC1 siRNA	Dharmacon	Cat#L-009290-00
SMARTpool ON-TARGETplus Human siRNA Rad18	Dharmacon	Cat#L-004591-00
SMARTpool ON-TARGETplus Non-targeting Control siRNAs #2	Dharmacon	Cat#D-001810-02
siGENOME Human HLTF siRNA-2	Dharmacon	Cat#D-006448-04
Recombinant DNA		
Plasmid: pcDNA4/TO	Invitrogen	Cat#V102020
Plasmid: pcDNA4/TO-PCNA-Puromycin	This paper	N/A
Plasmid: pcDNA4/TO-RFC1-Zeocin	This paper	N/A
Plasmid: pcDNA4/TO-HLTF-Zeocin	This paper	N/A
pSpCas9(BB)-2A-Puro (PX459) V2.0	Addgene	Cat#62988
Plasmid: PX459-HLTF-exon2	This paper	N/A
Plasmid: PX459-ZRANB3-exon2	This paper	N/A
Plasmid: PX459-SMARCAL1-exon1	This paper	N/A
Plasmid: PX459-MMS2-exon1	This paper	N/A
Plasmid: PX459-UBC13-exon2	This paper	N/A
Software and algorithms		
FlowJo 10.8	FlowJo, LLC	https://www.flowjo.com/
FIJI	NIH	RRID: SCR_002285
Prism 8	GraphPad	https://www.graphpad.com/scientificsoftware/prism/

### Experimental Model and Study Participant Details

#### Cell culture

The following cell lines used in this study (U2OS, hTERT-RPE1, HeLa Kyoto, HEK-293) were originally obtained from ATCC and were cultured in DMEM (Sigma). eHAP cells obtained from Horizon Discovery were cultured in IMDM medium (Life Technologies). All cultures were supplemented with 10% FBS (Invitrogen), L-glutamine (GlutaMAX, ThermoFisher) and occasionally with penicillin and streptomycin (Gibco). All cells were grown at 37°C in 5% CO_2_. The cell lines used in our study were authenticated by STR profilingand routinely tested for mycoplasma at the Crick Cell Services facility.

#### RNA interference, drugs, and small molecule inhibitors

Knockdown studies were performed using siRNA from Dharmacon. siRNAs were transfected at 5-50 nM final concentration depending on the experiment using Lipofectamine RNAiMAX (Invitrogen) and OptiMEM (Invitrogen). Experiments were completed 2 days after siRNA transfection. The following siRNAs were used: siRNA Control (siGENOME, non-targeting siRNA Pool #2, D-001206-14-20); siRNA HLTF-1 (ON-TARGETplus, SMARTpool, L-006448-00); siRNA HLTF-2 (siGENOME, D-006448-04); siRNA PCNA (ON-TARGETplus, SMARTpool, L-003289-00); siRNA RFC1 (ON-TARGETplus, SMARTpool, L-009290-00); siRNA ZRANB3 (siGENOME, D-010025-03-0010); siRNA SMARCAL1 (ON-TARGETplus, SMARTpool, L-013058-00-0005).The following used small molecule inhibitors/drugs were used: AZD7762 and MK-8776 (CHK1 inhibitors, 50-100 nM, Axon MedChem), VE-821 and VE-822 (ATR inhibitors, 500 nM, SelleckChem). Zeocin and blasticidin (4 μg/mL) were obtained from Life Technologies. Phalloidin (ThermoFisher). Aphidicolin (APH, 0.5-1 mg/mL), nocodazole (100 ng/mL), BrdU (20 μM) and cytochalasin B (4.5 μg/mL), PARPi (Olaparib), cisplatin, hydroxyurea and doxycycline were obtained from Sigma-Aldrich. Cdc7 inhibitor (XL-143, 5 to 10 μM, Axon), CDK2/1 inhibitor (roscovitine, 5 to 10 μM, Calbiochem), DNA-polymerase α inhibitors (CD437, Sigma-Aldrich and ST1926, MedChem).

### Cell lines

#### CRISPR-Cas9 knock-out cell lines

Tocreate of specific knockout cell lines, the following gRNA sequences were designed using Benchling software: (i) HLTF (Exon 2, GenBank NM_003071.3): 5' CACCGTTGGACTACGCTATTACAC 3' and 5' aaacGTGTAATAGCGTAGTCCAAC 3′.(ii) UBC13/UBE2N (Exon 2, GenBank NM_003348.4): 5' CACCGTAACGGGCGTTGCTCTCATC 3' and 5' aaacGATGAGAGCAACGCCCGTTAC 3′. (iii) MMS2/UBE2V2 (Exon 2, GenBank NM_003350.3): 5' CACCGTCCAACAAGCGAAAATTACG 3' and 5' aaacCGTAATTTTCGCTTG TTGGAC 3′. (iv) SHPRH (Exon 2, GenBank NM_001042683.3): 5' CACCGCTTCATTGGAATATGCATG 3' and 5' aaacCATGCA TATTCCAATGAAGC 3′. (v) ZRANB3 (Exon 2, GenBank NM_032143.4): 5' CACCGAGCTTTGCTCTTAGTCTGTC 3' and 5' aaacG ACAGACTAAGAGCAAAGCTC 3′. (vi) SMARCAL1 (Exon 3, GenBank NM_014140.4): 5' CACCGCGTAGTCAAATGGCTCTCAC 3' and 5' aaacGTGAGAGCCATTTGACTACGC 3′. In all cases, U2OS (or eHAP) parental cells were transfected using Lipofectamine 3000 (Invitrogen) with pX459-sgRNA (gRNAs targeting unique sequences within each gene locus) and selected with Puromycin for two days. Live single cells were sorted by flow cytometry, plated into 96-well plates, and allow to grow for 2 weeks. Individual clones were screened for lack of expression of the protein of interest by immunoblotting, using the specific antibodies detailed in the STAR Methods..

#### TetON cell lines

Stable TetON-HLTF cell lines were constructed by random plasmid integration. Human coding sequences of HLTF (GenBank NM_003071.3) were cloned using KpnI/NotI restriction enzymes into the pcDNA4/TO vector (Invitrogen) using the following primers:

Forward (KpnI) 5′GGGGTACCATGTCCTGGATGTTCAAGAGGG 3′

Reverse (NotI) 5′ATAGTTTAGCGGCCGCTTATAAGTCAATTAATGTTC 3′

T-REx-U2OS cells were obtained from Invitrogen. T-REx-HLTF-KO cells stably expressing the Tet repressor were constructed by transfecting HLTF-KO U2OS cells with the pcDNA6/TR plasmid (Invitrogen) and cells were selected in medium containing 5 μg/mL Blasticidin. Live single cells were sorted by flow cytometry, plated into 96-well plates, and allow to grow for 2 weeks. Individual clones were screened for the Tet repressor protein expression by immunoblotting with the specific antibodies (TET02, MoBiTec) and positive clones were selected. T-REx-wild type cells or T-REx-HLTF-KO cells were transfected with Lipofectamine 3000 (Invitrogen) using the pcDNA4/TO constructs carrying human HLTF sequence. Transformed cells were selected in 250 μg/mL Zeocin. Live single cells were sorted by flow cytometry, plated into 96-well plates, and allow to grow for 2 weeks. Individual clones were screened for Doxycycline dependent protein expression by immunoblotting with HLTF antibodies (ab17984, Abcam) and positive clones were selected.

For the generation of U2OS-tetON-PCNA and U2OS-tetON-RFC1, T-REx-U2OS cells were transfected with Lipofectamine 3000 (Invitrogen) using the pcDNA4/TO/Zeocin constructs carrying human PCNA (GenBank: NM_182649.2) or RFC1 (GenBank: NM_002913.5) coding sequence. Transformed cells were selected in 250 μg/mL Zeocin. Live single cells were sorted by flow cytometry, plated into 96-well plates, and allow to grow for 2 weeks. Individual clones were screened for Doxycycline dependent protein expression by immunoblotting with PCNA or RFC1 antibodies and positive clones were selected.The U2OS-tetON-PCNA-RFC1 cell line was generating by transfecting U2OS-tetON-RFC1 (Zeo) with pcDNA4/TO/Puromycin construct carrying human PCNA coding sequence.

## Method Details

### Flow cytometry and cell sorting

Multiplexed flow cytometry analysis using fluorescent cell barcoding, combined with EdU, antibody and DNA staining, was performed as previously described.^99^ Up to 6 samples subjected to different treatments were barcoded in each experiment to allow unbiased subsequent antibody staining of the combined samples. For detection of EdU, cells pulsed with 10 mM EdU for the indicated times were harvested and stained with Click-iT chemistry using Click-iT EdU Alexa Fluor 647 Flow Cytometry Assay Kit (Thermo, C10424) according to the manufacturer’s instructions. To quantify the amount of proteins in the insoluble/chromatin fraction, cells were treated with cytoskeleton buffer (CSK) 0.25% Triton X-100 for 5 min on ice. For DNA content analysis, cells were treated with 100 mg/mL RNase A and stained with 1 mg/mL DAPI. Data were analysed using FlowJo software. Cell doublets were excluded for all analyses. See Antibodies for details.

### Antibodies

The antibodies used for western blotting were the following: HLTF (ab17984, Abcam); pH2AX-S139 (γH2AX, 1:2000, clone JBW301, Millipore), pChk1-S345 (1:3000, 2348L, Cell signalling); pKAP1 S824 (1:1000, A300-767A, Bethyl); pRPA2-S4S8 (1:7500, A300-245A, Bethyl); KU70 (1:3000, sc-5309, Santa Cruz); alpha-Tubulin (1:4000, Sigma, T5168); PCNA (1:1000, PC10, Santa Cruz); RFC3 (1:1000, ab182143, Abcam); RFC1 (1:1000, ab229229, Abcam); RFC1 (1:1000, sc-271656, Santa Cruz); H3 (1:1000, ab1791, Abcam); SMARCAL1 (1:2500, sc-376377, Santa Cruz); ZRANB3 (1:500; Proteintech); MMS2 (1:1000, ab155007, Abcam); UBC13 (1:1000, 4E 11, ThermoFisher); SHPRH (1:1000, ab80129, Abcam). Blots were imaged using the LI-COR platform (LI-COR Biosciences) with anti-rabbit RDye 800CW and anti-mouse IRDye680RD secondary antibodies (both 1:10000). Images were analyzed and quantified using the Odyssey Infrared imaging system (LI-COR Biosciences).

The antibodies used for flow cytometry were the following: pH3-S10 (1:200, ab14955, Abcam); pH2AX-S139 (γH2AX, 1:250, clone JBW301, Millipore); PCNA (1:250, PC10, Santa Cruz); RPA32 (1:250, ab2175, Abcam); RPA70 (1:250, EPR3472, Abcam). For chromatin RPA exhaustion, pH2AX-S139 (γH2AX, 1:250, ab11174, Abcam) in combination with RPA32 (1:250, ab2175, Abcam). The antibodies used for immunofluorescence studies were the following: RPA (Ab-2 NA18, Calbiochem), RPA (9H8 ab2175, Abcam); Rad51 (H92, Santa Cruz); BrdU (Becton, Dickinson).

### DNA fiber stretching assay

U2OS cells were pulse labelled with 25 μM CldU for 20 min, washed with PBS and subsequently pulse labelled with 250 μM IdU together with APH (1 mg/mL) with or without Chk1 inhibitor (AZD7662, 50nM) or ATR inhibitor (VE-821, 500 nM) for the indicated time (from 10 min to 240 min). Cells were washed with warm PBS three times and immediately harvest or release in complete media with 250 μM IdU for the indicated time (20 to 120 min). Cells were then trypsinised and resuspended at a concentration of 4×10^5^ cell/mL in cold PBS. 3 μL of cell suspension were spotted on clean glass slides, air-dried for 5 min and lysed with 8 μL of 0.5% SDS in 200 mM Tris-HCL, pH 7.4, 50 mM EDTA (2-5 min, RT). Slides were slightly tilted, allowing the drop to run down the slide, air-dried for 10 min and then fixed in fresh methanol/acetic acid (3:1) for 10 min at RT. Then, air dried ON at 4 °C. Samples were then denatured (2.5 M HCl for 75 min). Slides were blocked in 1% BSA/PBS + 0.1% Tween-20 for 30 min at RT and incubated with rat anti-BrdU monoclonal antibody (αCldU; AbD Serotec) and mouse anti-BrdU monoclonal antibody (αIdU; Becton Dickinson), both 1:500 for 1 h at RT. Later, slides were incubated with anti-rat IgG Alexa Fluor 488 (Green, Molecular Probes) and anti-mouse and IgG AlexaFluor 555 (Red, Molecular Probes) both at 1:500 for 1.5 h at RT. Slides were washed and mounted with Fluoroshield (Sigma). Images were acquired using a Zeiss Upright 710 confocal microscope with 60x or 43x oil immersion objective. Fiber length was measured using FIJI. The pixel size values were converted into micrometres (μm) using the scale bars created by the microscope and then converted to kilobases according to the conversion factor 1 μm = 2.59 kb. For IdU length measures, otherwise indicated, only bicolour fibers were counted (green-red). For new origin firing quantification, origins fired during the second pulse (IdU, red structures) were quantified as percentage of all structures containing red (green-red and red only). Between 200-700 total replication structures were scored in each sample. Experiments were performed in duplicate or in triplicate. DNA fiber data are presented as box-and-whisker plots, with the median shown as a thick line, boxes representing the interquartile range (25th–75th percentiles), and whiskers indicating the 10th–90th percentile range. All statistical comparisons of DNA fiber tract lengths were performed using the two-tailed Mann–Whitney U test (nonparametric, unpaired) where the statistical significance was defined as *p < 0.05, **p < 0.01, ***p < 0.001, and **p < 0.0001. ns indicates non-significant differences.

### Nuclear aberration analysis

Transfected cells were replated at low density. 24 h after replating cytochalasin B (4.5 μg/mL Sigma) was added to the media and 40 h later cells were washed 1 min with hypotonic buffer (KCl 0.0075 M) diluted 1/10 from stock solution in PBS 1X, twice with PBS 1X and fixed with PFA/sucrose 2% for 20 min. Phalloidin and DAPI staining were used to visualize the whole cell and its nuclei respectively.

### Survival Assay

For colony formation assays, cells were seeded in duplicates (6-cm dishes) per condition and allowed to adhere for a minimum of 6 h. Cells were then treated with indicated concentrations of APH for 24 h, washed extensively and incubated in drug-free medium for 11 days. Plates were then washed once in PBS, left to dry, and stained with cell staining solution (0.5% w/v crystal violet, 25% v/v methanol). Finally, the plates were washed three times in deionized water. Colonies were counted manually and surviving fraction calculated as: colonies/(seeded colonies × plating efficiency). For cell viability assays, we used CellTiter-Glo Luminescent Cell Viability Assay (Promega) which measures total cellular ATP. 200 to 500 cells per well were seeded in white opaque 96-well plates. 24 h later, cells were treated with the indicated concentrations of APH, checkpoint inhibitors, HU, PARP inhibitors for 6 days. Prior to measurement, 75 μL of CellTiter-Glo reagent was added into each well and the contents were mixed for 30 min to induce cell lysis on an orbital shaker. Luminescence was measured using the bioluminescence plate reader Envision 2102 (Perkin – Elmer).

### Immunofluorescence staining

#### Anti-BrdU

To detect single stranded DNA under non-denaturing conditions, U2OS cells were seeded 24 h prior to treatment on 12 mm coverslips in 12-well plates. For parental ssDNA, BrdU (10 μM) was added for a total of 48h (24h before APH treatment and 24h with APH treatment). For nascent ssDNA, BrdU (20 μM) was added to APH-treated cells 10 min before and during the 4 h treatment with APH +/-checkpoint inhibitors. Cells were permeabilised at 4°C for 10 min in PBS + 0.5% Triton X-100 followed by fixation at room temperature (RT) for 10 min in 4% paraformaldehyde (PFA). Cells were fixed again at -20°C with 100% methanol for 10 min, dried for 1 min, and then washed with PBS. Cells were then incubated at RT with blocking solution -PBS-T and 2% BSA-for 1h. Coverslips were then incubated with primary antibodies: mouse anti-BrdU (1:200, BD Biosciences; clone B44) at 37°C for 1 h. Coverslips were washed for 5 min in PBS-T and incubated at RT for 1 h with secondary antibodies (1:500; Alexa Fluor 488). Coverslips were then washed with PBS-T and stained with DAPI (1 μg/mL) and mounted on microscope slides with Fluoroshield (Sigma).

#### RPA and Rad51

U2OS cells were seeded on 12 mm coverslips in 12-well plates 24 h prior to treatment. Cells were treated with the indicated drugs. Coverslips were then incubated in cytoskeleton buffer (CSK) 0.5% Triton X-100 for 5 min on ice and fixed with 4% PFA for 20 min at RT. Coverslips were then washed twice in PBS, permeabilized with 0.1% Triton X-100 in PBS for 15 min at 4°C and blocked in 5% BSA 0.1% Triton X-100 in PBS overnight. Primary incubation was performed overnight in blocking solution using the anti-RAD51 antibody (1:100, Abcam; ab133534). Coverslips were then washed with PBS containing 0.25% Tween 20. Secondary incubation was performed in PBS using Alexa 488 (1:500) for 1 h at RT. Coverslips were then washed with PBS-T and stained with DAPI (1 μg/mL) and mounted on microscope slides with Fluoroshield (Sigma). Statistical comparisons of IF intensity were performed using the two-tailed Mann–Whitney U test (*p < 0.05, **p < 0.01, ***p < 0.001, and **p < 0.0001).

#### Neutral Comet Assay

Neutral comet assays were performed using a Comet Assay kit from Amsbio (4250-050-K) according to the manufacturer’s protocol.

#### Cell fractionation

1×10^6^ U2OS cells were seeded. 24 h later cells were treated for 24 h with APH alone or in combination with checkpoint inhibitors (AZD-7662, 100nM) before harvesting for the indicated times. Cells were scraped in 1 mL ice-cold PBS and spun down for 4 min at 500 g and resuspended in 200 μL CSK buffer (10mM PIPES pH7.0, 100mM NaCl, 300mM Sucrose, 3 mM MgCL2, 1mM EGTA, 0.5% Triton, 1mM DTT and 1x of protease inhibitor mix (Complete, EDTA-free, Roche). Samples were incubated on ice for 10 min. Cells were spun down for 3 min at 1000 rpm. 150 μL of supernatant, representing the soluble fraction, was collected and 50 μL of 4x Laemmli Buffer (Bio-Rad) + 10% 2-metcaptoethanol (Sigma-Aldrich) was added. Residual soluble fraction was removed, and the chromatin pellet was washed in 500 μl of CSK. 200 μL of 1x Laemmli Buffer + 2.5% 2-metcaptoethanol was added. Samples were sonicated with a probe at medium intensity (10A) for 12 s in a Branson 250 instrument, and then incubated at 95°C for 10 min. 20 μL of each sample was loaded and subjected to SDS-PAGE and immunoblotting.

### Quantification and Statistical Analysis

Statistical analysis was performed using the Mann–Whitney U test (two-tailed) to compare two independent groups, using Prism (GraphPad Software). Significance levels are indicated as follows: n.s. (not significant), * (*p* < 0.05), ** (*p* < 0.01), *** (*p* < 0.001), **** (*p* < 0.0001). For DNA fiber analysis, data are displayed as box-and-whisker plots, where the box represents the interquartile range (25th–75th percentiles), the thick line indicates the median, and whiskers show the 10th–90th percentile range. Statistical comparisons of DNA fiber tract lengths were performed using the Mann–Whitney U test (two-tailed), with significance thresholds as described above.

## Supplementary Material

Supplemental information can be found online at https://doi.org/10.1016/j.molcel.2025.06.002.

Supplementary Information

Table S1

Table S2

## Data Availability

Original data images have been deposited at Mendeley and are publicly available as of the date of publication. Mendeley data: https://doi.org/10.17632/9k5cnmrvxk.1. This study does not report original code. Any additional information required to reanalyze the data reported in this paper is available from the lead contact upon request.

## In brief

The DNA replication checkpoint prevents continued origin firing and fork collapse after replication fork stalling. Bertolin et al. show that excess Okazaki fragments from continued origin firing sequester replication processivity factors RFC and PCNA, leading to deprotection of DNA ends and fork collapse driven by the HLTF fork remodeler.

## Highlights

- The DNA replication checkpoint prevents accumulation of excess Okazaki fragments
- Excess Okazaki fragments deplete PCNA/RFC1 from replication forks
- PCNA/RFC1 depletion deprotects DNA ends, allowing enzymatic attack
- HLTF drives replication fork collapse from deprotected ends in human cells
