# Supplementary material for: The DNA replication checkpoint prevents PCNA/RFC depletion to protect forks from HLTF-induced collapse in human cells
Source: Mol Cell. Author manuscript; Available in PMC 2026 Aug 26. (PMC7619400; doi:10.1016/j.molcel.2025.06.002)
Supplement: Supplementary file 1 [file EMS217826-supplement-Supplementary_Information.pdf]

Molecular Cell, Volume *85*

Supplemental information

The DNA replication checkpoint prevents PCNA/RFC depletion to protect forks from HLTF-induced collapse in human cells

Agostina P. Bertolin, Berta Canal, Mona Yekezare, Anne Early, Jingkun Zeng, Rachael Instrell, Michael Howell, and John F.X. Diffley

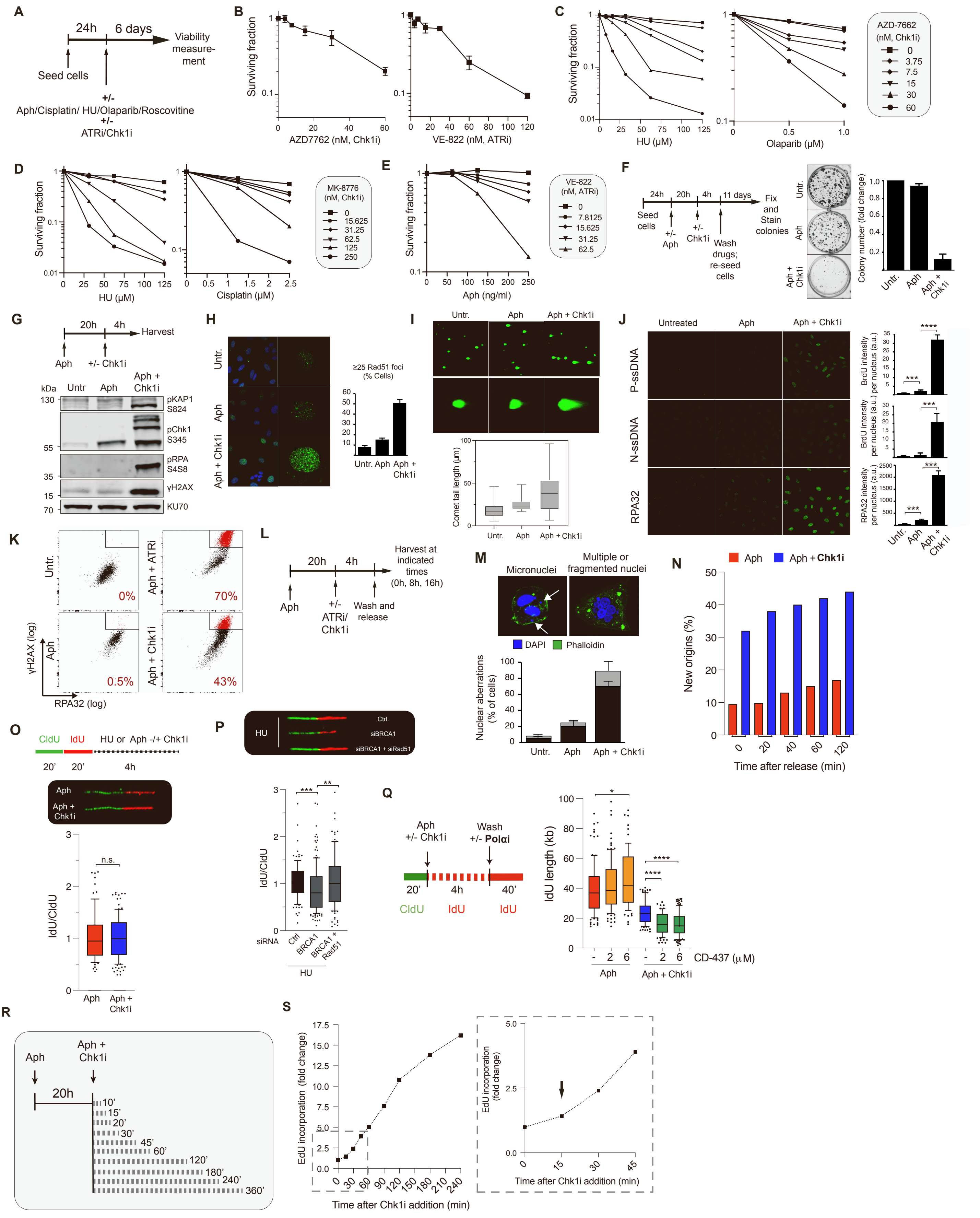

A
24h
6 days
Viability measurement
Seed cells
+/-
Aph/Cisplatin/ HU/Olaparib/Roscovitine
+/-
ATRi/Chk1i
B
Surviving fraction
AZD7762 (nM, Chk1i)
VE-822 (nM, ATRi)
C
HU (μM)
Olaparib (μM)
AZD-7662 (nM, Chk1i)
0
3.75
7.5
15
30
60
D
HU (μM)
Cisplatin (μM)
MK-8776 (nM, Chk1i)
0
15.625
31.25
62.5
125
250
E
Aph (ng/ml)
VE-822 (nM, ATRi)
0
7.8125
15.625
31.25
62.5
F
Seed cells
+/- Aph
+/- Chk1i
Wash drugs; re-seed cells
Fix and Stain colonies
Colony number (fold change)
Untr.
Aph
Aph + Chk1i
G
20h
4h
Harvest
Aph
+/- Chk1i
kDa
Untr
Aph
Aph + Chk1i
pKAP1 S824
pChk1 S345
pRPA S4S8
γH2AX
KU70
H
≥25 Rad51 foci (% Cells)
I
Comet tail length (μm)
J
Untreated
Aph
Aph + Chk1i
P-ssDNA
N-ssDNA
RPA32
BrdU intensity per nucleus (a.u.)
RPA32 intensity per nucleus (a.u.)
K
Untr.
Aph + ATRi
Aph
Aph + Chk1i
0%
70%
0.5%
43%
γH2AX (log)
RPA32 (log)
L
20h
4h
Harvest at indicated times (0h, 8h, 16h)
Aph
+/- ATRi/ Chk1i
Wash and release
M
Micronuclei
Multiple or fragmented nuclei
DAPI
Phalloidin
Nuclear aberrations (% of cells)
Untr.
Aph
Aph + Chk1i
N
Aph
Aph + Chk1i
New origins (%)
Time after release (min)
O
CldU
IdU
HU or Aph -/+ Chk1i
20'
20'
4h
IdU/CldU
n.s.
P
HU
Ctrl.
siBRCA1
siBRCA1 + siRad51
siRNA
Ctrl
BRCA1
BRCA1 + Rad51
Q
Aph +/- Chk1i
Wash +/- Polα
20'
4h
40'
CldU
IdU
IdU
IdU length (kb)
CD-437 (μM)
Aph
Aph + Chk1i
R
Aph
Aph + Chk1i
20h
10'
15'
20'
30'
45'
60'
120'
180'
240'
360'
S
EdU incorporation (fold change)
Time after Chk1i addition (min)

Figure S1. Checkpoint inhibition after replication fork stalling induces S phase catastrophe, related to Figure 1 and Figure 2.

(A) Experimental setup for cell viability assays using CellTiter-Glo.
(B-E) U2OS cells were treated as indicated in **Figure S1A** with checkpoint inhibitors (ATRi, VE-822; Chk1i, AZD7662 and MK-8776) alone or in combination with different genotoxic drugs at the indicated concentrations. Cell viability was normalised to cells untreated with genotoxic drugs for each checkpoint inhibitor condition.
(F) Clonogenic survival of U2OS cells. Left panel, experimental set up. After drugs were washed off, cells were released in complete media and allowed to grow for 14 day. Colonies were then fixed and stained with crystal violet. Middle panel, representative images of colony staining. Right panel, quantification showing the fold change in colony number relative to untreated condition. Error bars represent SEM.
(G) Western blot analysis of DNA damage markers following treatment depicted in the upper panel. The following DNA damage markers are used throughout this study: (i) γH2AX is phosphorylated by DNA-PK, ATR, and ATM, and is therefore considered a general marker of replication stress and DNA damage; (ii) pKAP1-S824 and pRPA-S4/S8 are primarily phosphorylated by DNA-PK and are typically associated with DNA double-strand breaks (DSBs);(iii) pCHK1-S345 is specifically phosphorylated by ATR and serves as a proxy for replication stress. KU70 protein levels were used as loading controls.
(H) Immunofluorescence analysis of chromatin-bound Rad51. Right panel, representative images. Left panel, graph showing percentage of cells with equal or more than 25 Rad51 foci.
(I) Assessment of DNA strand break formation by neutral comet assay. Upper panel, representative comets. Bottom panel, quantification of comet length. At least 50 comets were scored per sample.
(J) Immunofluorescence analysis of parental and nascent single strand DNA (P-ssDNA and N-ssDNA) and chromatin-bound RPA32. Left panel, representative images. Right panel, quantifications.
(K) Flow cytometry analysis of the levels of chromatin-bound RPA32 and γH2AX in the same population of cells. Cells were left untreated or treated with 1 μg/ml APH alone or in combination with checkpoint inhibitors (ATRi, VE-822; Chk1i, AZD7662). Representative images comparing γH2AX intensity (log scale) and chromatin-bound RPA32 intensity (log scale) in cells. Gated cells depicted in red represent cells with simultaneously high levels of γH2AX and RPA. Bottom right of each panel, quantification of the percentage of red coloured cells.
(L) Experimental setup used for **Figures 1B**, **1C** and **S1M**. Cells were seeded and, after 24 h, left untreated or treated with 1 μg/ml APH alone or in combination with 50 nM Chk1 inhibitor (AZD7662). Following treatment, cells were immediately harvested, or drugs were washed, and cells were released in complete media which depending on the experiment contained no drug, 100 nM taxol or

4.5 μg/ml cytochalasin B.

(M) Genomic instability analysis. U2OS cells were treated like **Figure 1L** but analysed forty hours after release in complete media with 4.5 μg/ml cytochalasin B. Cells then were fixed, stained with DAPI and phalloidin and analyzed for nuclear aberrations. Upper panel, representative images. Bottom panel, graph showing percentage of cells showing nuclear aberrations.

(N) Percentage of new origins fired in samples shown in **Figures 1D-1F** was determined by counting the number of IdU/red-only fibers over the total number of different replication structures. At least 600 of total structures were scored per sample.

(O) Nascent DNA resection assay in cells treated with APH and APH/Chk1. Upper panel, experimental setup for Fig S1O and P. Cells were pulsed with CldU for 20 min, washed, then incubated with IdU for 20 min, washed and treated with HU (4 mM) or 1 μg/ml APH alone or in combination with 50 nM Chk1i for 4h. Middle panel, representative micrographs of CldU and IdU replication tracks. Bottom panel, box-and-whisker plot and median IdU track length in bicolour fibers. At least 100 forks were scored per sample.

(P) Nascent DNA resection assay in cells treated with HU (4 mM). Upper panel, representative micrographs of CldU and IdU replication tracks. Bottom panel, box-and-whisker plot and median IdU track length in bicolour fibers. At least 150 forks were scored per sample.

(Q) DNA fiber analysis of replication fork progression. Left panel, experimental setup: cells were pulsed with CldU for 20 min, washed and then incubated with IdU plus 1 μg/ml APH alone or in combination with 50 nM Chk1i for 4h. Cells were then washed and released in complete media containing IdU and the Polα inhibitor CD-437 for 40 min. Right panel, box-and-whisker plot and median IdU track length in bicolour fibers is shown. At least 100 forks were scored per sample.

(R) Timing of events following checkpoint inhibition. Schematic of the experimental approach used in **Figures 2A**, **2B** and **S2C**. U2OS cells were treated with 1 μg/ml APH for 20 h and then Chk1i (AZD7662, 50 nM) was added for the indicated times.

(S) EdU incorporation of cells treated with Chk1i at different times. Graphs show the fold change in EdU mean fluorescence intensity (MFI) at each time point relative to APH treatment alone. Right panel, graph inset for 0-45 min of Chk1i treatment.

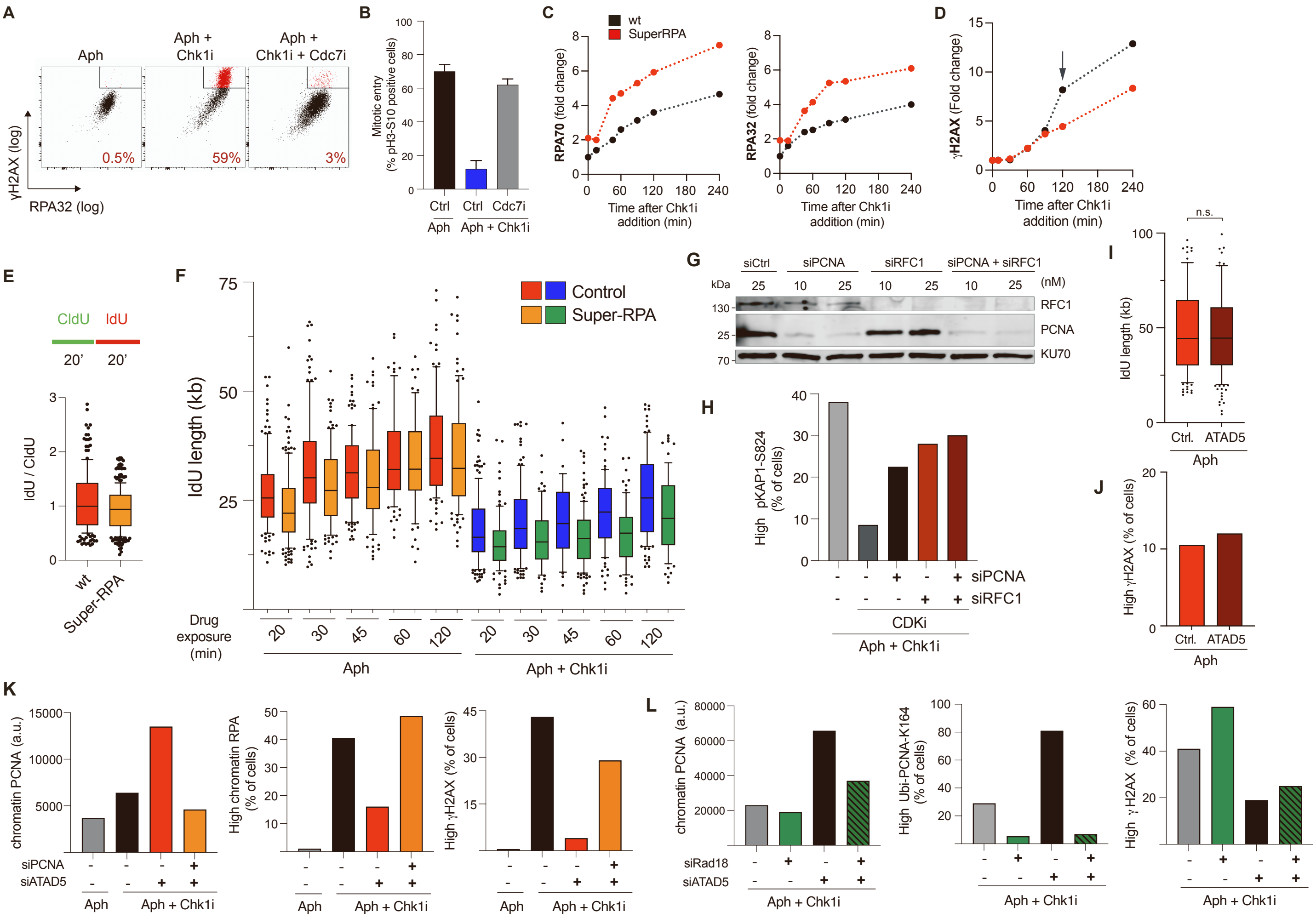

Figure S2. Excess origin firing depletes chromatin-bound PCNA and causes replication fork defects, related to Figure 3.

(A, B) Inhibition of new origin firing rescues checkpoint inhibition phenotypes. Cells were pre-treated with Cdc7i (XL143, 10 μM) for 60 minutes prior to further treatment . **(A)** Flow cytometry analysis of chromatin-bound RPA32 and γH2AX levels in the same population of cells. Cells were treated with APH alone and APH/Chk1i alone or in combination with Cdc7i (10 μM). Representative images comparing γH2AX intensity (log scale) and chromatin-bound RPA32 intensity (log scale) in treated cells. Gated cells depicted in red represent cells with simultaneously high levels of γH2AX and RPA. Right bottom corner, quantification of the percentage of red coloured cells in each sample. **(B)** Mitotic entry analysis. Cells were treated like Figure S1L and 16 hours after release in complete media with 100 nM taxol, cells were fixed and stained for pH3-S10 and DAPI followed by flow cytometric analysis. The proportion of pH3-S10 positive cells in each sample is shown.

(C-F) Overexpression of RPA complex using the SuperRPA cell line does not rescue early phenotypes of checkpoint inhibition. Flow cytometry analysis of chromatin-bound RPA70, RPA32 **(C)** and γH2AX **(D)** in wild-type and SuperRPA cells treated with APH and Chk1i for different times.

Graphs show the fold change in mean fluorescence intensity (MFI) of each protein at each time point relative to APH treatment alone. **(E)** DNA fiber analysis of replication fork progression in unperturbed conditions in wild-type and SuperRPA cells. Cells were pulsed with CldU for 20 min, washed, then incubated with IdU for 20 min. Box-and-whisker plot and median IdU track length in bicolour fibers is shown. At least 200 forks were scored per sample. **(F)** DNA fiber analysis of replication fork progression in wild-type and SuperRPA cells. The experimental set up used is depicted in **Figure 2C**. Cells were pulsed with CldU for 20 min, washed and then incubated with IdU plus 1 μg/ml APH alone or in combination with 50 nM Chk1i for the indicated amount of time. Cells were then washed and released in complete media containing IdU for 40 min. Box-and-whisker plot and median IdU track length in bicolour fibers is shown. At least 200 forks were scored per sample.
(G) Immunoblots of whole-cell extracts from U2OS cells depleted of PCNA, RFC1, or both, using siRNA at 10 nM and 25 nM under unperturbed conditions. KU70 served as a loading control.
(H) Flow cytometry analysis of cells with high pKAP1-S824 levels after treatment with APH and Chk1i, with or without the CDK inhibitor roscovitine, following depletion of PCNA, RFC1, or both.
(I) DNA fiber analysis of replication fork progression in ATAD5-depleted cells following 24 h APH treatment. Box-and-whisker plots show IdU track lengths from bicolour fibers. At least 150 forks were scored per condition.
(J) Flow cytometry analysis of the percentage of control and ATAD5-depleted cells exhibiting high γH2AX staining after 24 h of APH treatment.

(K) Flow cytometry analysis of the chromatin-bound PCNA, chromatin-bound RPA and γH2AX levels. Control cells were treated with APH alone and control cells and cells depleted for ATAD5 alone or in combination with PCNA were treated with 1 μg/ml APH for 24h, with the addition of 50 nM Chk1 inhibitor during the final 4 hours.
(L) Flow cytometry analysis of the chromatin-bound PCNA, ubiPCNA-K164 and γH2AX levels. Control cells and cells depleted for ATAD5 alone or in combination with Rad18 were treated with 1 μg/ml APH for 24 h, with the addition of 50 nM Chk1 inhibitor during the final 4 hours.

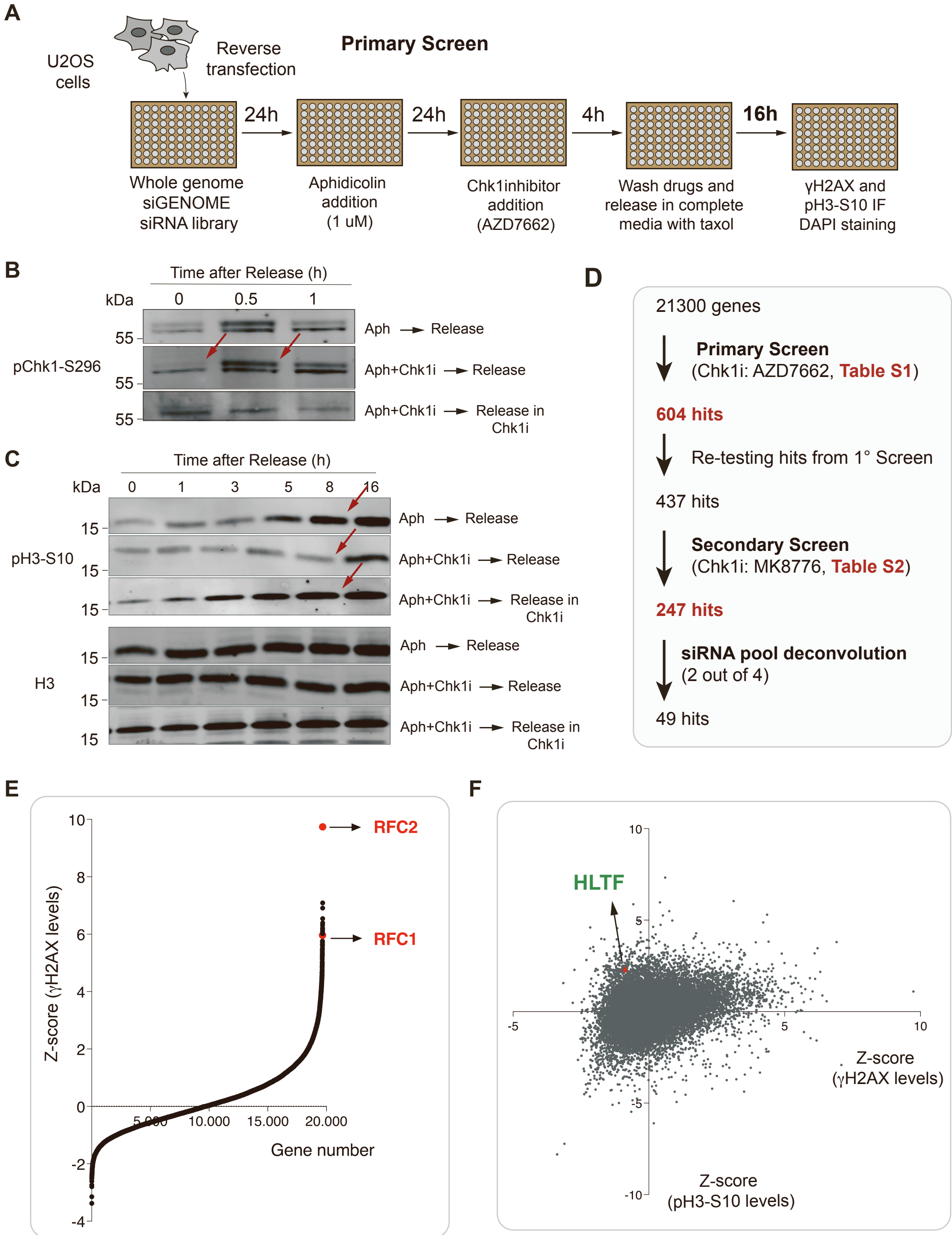

Figure S3. Whole-genome siRNA screen to identify genes whose depletion modulates, either enhances or suppresses, DNA damage and mitotic entry induced by checkpoint inhibition, related to Figure 4.

(A) Overview of the RNA interference-based genome-wide screen methodology. U2OS cells were plated in 96 well plates and reversed transfected with siRNAs. Twenty-four hours later, cells were treated with APH for 24 h and then a reversible Chk1 inhibitor (50nM, AZD7762) was added for 4 h.

APH and Chk1 inhibitor were then removed, and cells were released in medium containing taxol for 16 h. Cells were harvested, stained for γH2AX and pH3-S10, and fluorescence intensity values for both markers were measured by automated fluorescence microscopy at the Crick High Throughput Screening Laboratory.

(B) Chk1 reactivation after transient inhibition with Chk1 inhibitor occurs at around 30 min after inhibitor wash out (red arrows). Immunoblot of U2OS cells showing phosphorylation of Chk1-S296 (Chk1 auto-phosphorylation). Cells were treated with 1 μg/ml APH for 24h, with the addition of 50 nM Chk1i (AZD7662) during the final 4 hours. Drugs were then washed out and cells were released in complete media with or without Chk1i. Samples were analysed at 0, 30, and 60 minutes after release.

(C) Chk1 reactivation following transient inhibition with the Chk1 inhibitor triggers G2/M arrest, as indicated by the delayed appearance of H3-S10 phosphorylation (pH3-S10, a mitotic marker; red arrows). Immunoblot of U2OS cells showing phosphorylation of H3-S10. Cells were treated with 1 μg/ml APH for 24 h, with the addition of 50 nM Chk1i (AZD7662) during the final 4 hours. Drugs were then washed out and cells were released in complete media with 100 nM of taxol with or without Chk1i for 1, 3, 5, 8 and 16 hours.

(D) Workflow diagram of the sequential siRNA screens. The primary screen was performed independently twice and identified 437 overlapping siRNAs that reduced γH2AX and increased pH3-S10 levels by at least 1.5 standard deviations from the population mean (**Table S1**). Those 437 siRNAs were re-tested in a secondary screen under the same conditions as the primary screen, but using a different Chk1 inhibitor (MK-8776), yielding a total of 247 siRNAs (**Table S2**). siRNAs were considered for further study when they simultaneously caused a decrease in γH2AX levels, indicating reduced DNA damage, and an increase in pH3-S10 levels, indicating enhanced mitotic entry. This criterion was chosen to detect siRNAs that allow a 'functional' rescue of the irreversibility of replication arrest, as not only DNA damage was monitored but also, and most importantly, the ability of these forks to resume replication, complete S phase, and enter mitosis. Next, the top 121 scoring siRNA pools were re-screened by siRNA deconvolution. For each selected pool, the four individual siRNAs comprising it were tested separately for their ability to increase pH3-S10 levels under the same conditions. Forty-nine genes showed a clear increase in mitotic index (≥ 30% compared to control) with at least 2 out of 4 siRNA oligos.

(E) Primary screen Z-score distribution of all siRNAs analysed for DNA damage assessed by γH2AX levels. Each dot represents the average Z score of an individual siRNA tested in triplicate. Out of the 21,000 genes tested, RFC1 depletion ranked among the top 20, while RFC2 depletion was the highest-scoring gene.

(F) Primary screen Z-score distribution of all siRNAs analysed for DNA damage (γH2AX) and mitotic entry (pH3-S10). One of the strongest hits for both parameters was helicase-like transcription factor

(HLTF), a RING domain-containing ubiquitin ligase and SNF2-family DNA translocase previously implicated in several DNA repair and tolerance pathways.

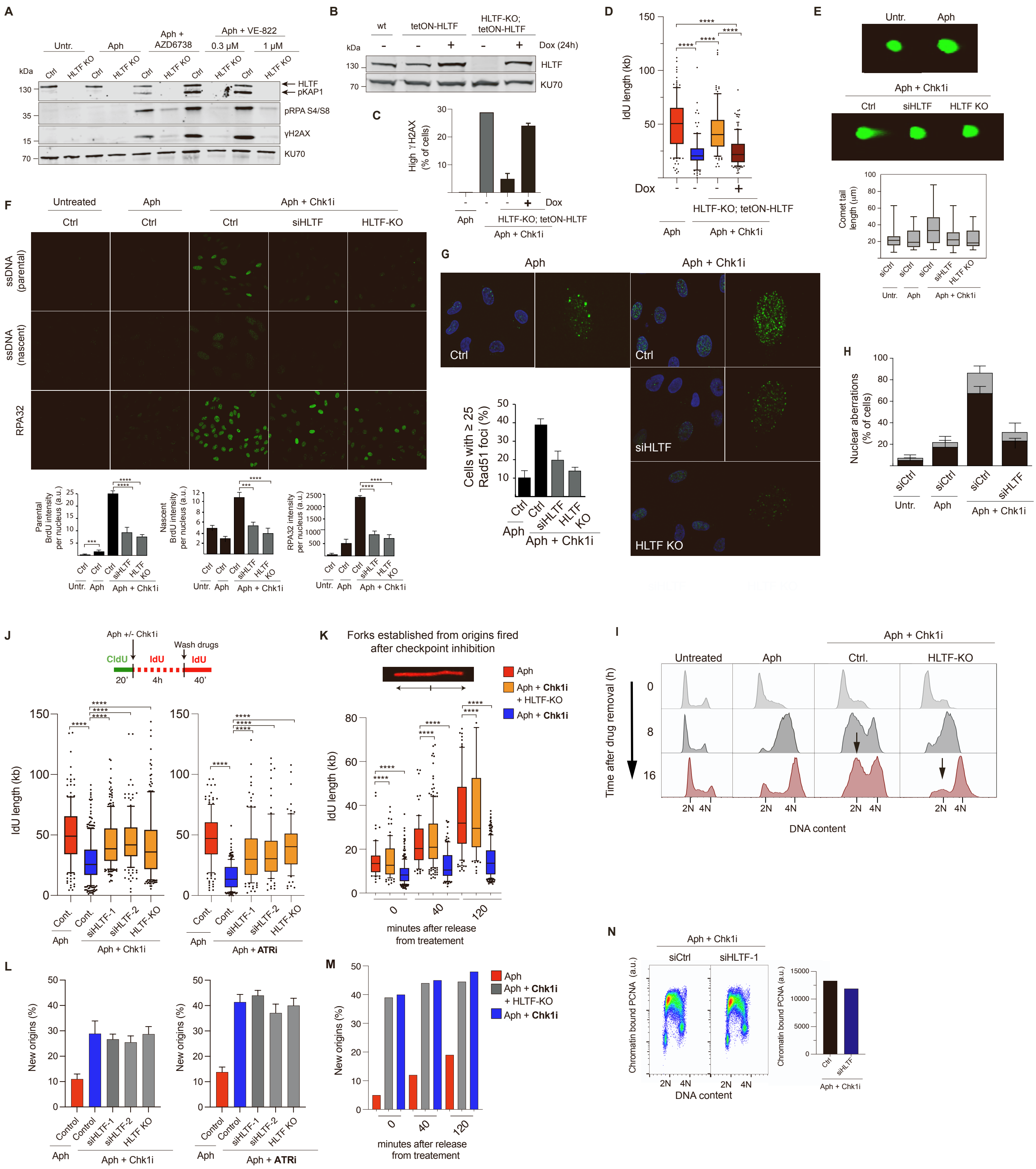

Figure S4. HLTF is responsible for the defects caused by the absence of the checkpoint, related to Figure 4.

(A) Immunoblots showing of DNA damage markers (pKAP1, pRPA-S4S8 and γH2AX) in control and HLTF CRISPR knockout (KO) U2OS cells after ATR inhibition. Control and HLTF-KO cells were left

untreated, treated with 1 µg/ml APH for 24 h, or with APH for 24 h combined with ATR inhibitors (AZD6738 or VE-822) during the final 4 h. Cells were harvested immediately after treatment. KU70 was used as a loading control.

(B) Immunoblots showing doxycycline (Dox)-induced expression of wild-type HLTF in tetON-HLTF cells generated in a wild-type U2OS background (left) or an HLTF-KO background (right).

(C) Flow cytometry analysis of the percentage of cells presenting high levels of γH2AX staining. Control cells were treated with 1 µg/ml APH for 24h alone and control, HLTF-KO cells and HLTF-KO; tetON-HLTF were treated with APH for 24h and a checkpoint inhibitor was added during the final 4 hrs. After treatment, cells were immediately harvested.

(**D**) DNA fiber analysis of replication fork progression in HLTF-KO; tetON-HLTF cells following 24 h doxycycline induction. Box-and-whisker plots show IdU track lengths from bicolour fibers. At least 200 forks were scored per condition.

(E) Assessment of DNA strand break formation by neutral comet assay. Upper panel, representative comets. Bottom panel, quantification of comet length. At least 40 comets were scored per sample.

(F) Immunofluorescence analysis of parental and nascent single strand DNA, chromatin-bound RPA32 in control, HLTF knock down (siHLTF) and HLTF-KO cells treated like **Figure S1G**. Upper panel, representative images. Bottom panel, quantification of BrdU or RPA32 intensity.

(G) Immunofluorescence analysis of chromatin-bound Rad51 in siHLTF and HLTF-KO cells after checkpoint inhibitor treatment. Left panel, graph showing percentage of cells with equal or more than 25 Rad51 foci. Right panel, representative images.

(H) Genomic instability in control and HLTF deficient cells. Control cells were left untreated or treated with 1 µg/ml APH for 24h alone. Control and siHLTF treated cells were treated with APH for 24h and a checkpoint inhibitor was added during the final 4 hrs. Forty hours after release in complete media containing 4.5 µg/ml cytochalasin B, cells were fixed, stained with DAPI and phalloidin and analyzed for nuclear aberrations. Graph shows percentage of cells showing nuclear aberrations.

(I) Flow cytometry analysis of S phase progression. Control cells were left untreated or treated with 1 µg/ml APH for 24h alone. Control and HLTF-KO cells were treated with APH for 24h and a checkpoint inhibitor was added during the final 4 hrs. 0h, 8h and 16h after release in complete media with 100 nM taxol, cells were fixed and stained with DAPI followed by flow cytometric analysis. Cell cycle histograms are shown. Black arrows indicate the S-phase progression defect induced by checkpoint inhibition, which is rescued in HLTF-KO cells.

(J-L) DNA fiber analysis of replication fork progression and origin firing in HLTF-KO and HLTF-depleted (siHLTF) U2OS cells. (**J**) Upper panel, experimental set up: cells were pulsed with CldU

for 20 min, washed and then incubated with IdU plus 1 μg/ml APH alone or in combination with Chk1i (AZD7662, 50 nM) or ATRi (VE-822, 500 nM) for 4h. Cells were then washed and incubated again only with IdU for 40 min. Bottom panel, box-and-whisker plot and median IdU track length in bicolour fibers. At least 150 forks were scored per sample.

(K) Box-and-whisker plot showing the median IdU track length in red-only fibers (representing forks fired after drug treatment) from the samples shown in **Figures 4D** and **4E**.

(L) Percentage of new origins fired measured by DNA fiber analysis in samples shown in **Figure S5J**.

(M) Percentage of new origins fired measured by DNA fiber analysis in samples shown in **Figures 4D** and **4E**. The percentage of new origins fired during checkpoint inhibition was determined by counting the number of IdU/red-only fibers over the total number of different replication structures. At least 600 of total structures were scored per sample.

(N) Flow cytometry analysis of chromatin-bound PCNA levels in control or HLTF-depleted cells treated with APH and Chk1 inhibitor, as described in **Figure S5A**.

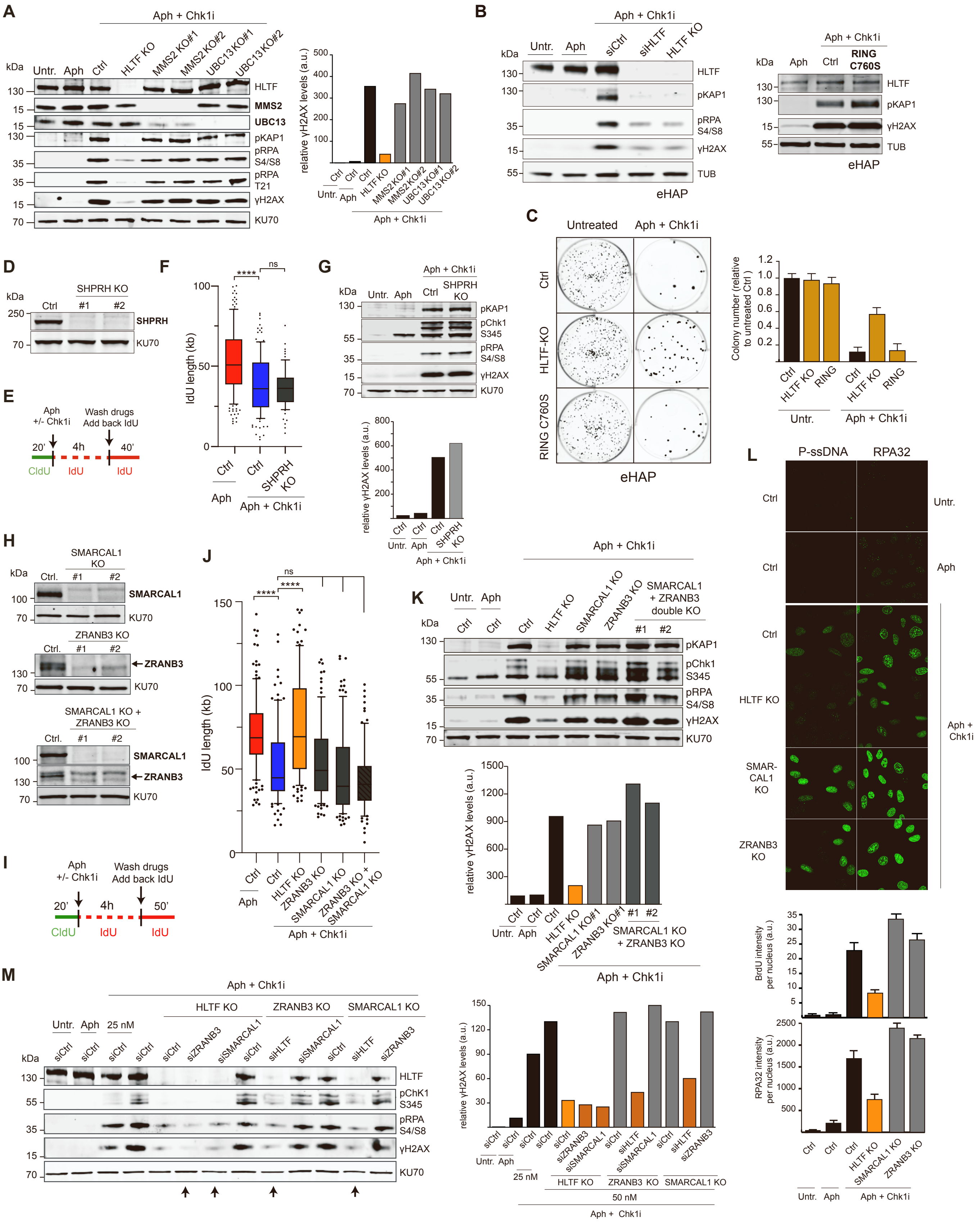

Figure S5. Defects caused by checkpoint loss are not mediated by key factors of the PCNA polyubiquitination pathway or the fork reversal translocases ZRANB3 and SMARCAL1,

related to Figure 4.

(A) MMS2/UBC13 PCNA, heterodimeric E2 ubiquitin conjugatin enzymes involved in the PCNA polyubiquitylation pathway, were individually knocked out in U2OS cells. DNA damage was monitored by immunoblotting of pKAP1, pRPA-S4/S8, pRPA-T21 and γH2AX, in cells treated with aphidicolin (APH) +/- Chk1 inhibitor. Right panel, quantification of γH2AX intensity from the blot.
(B) Immunoblots of HLTF and DNA damage markers (pKAP1, pRPA-S4/S8, and γH2AX) following Chk1 inhibition in eHAP cells. Left panel, HLTF knockdown (siHTLF) and knockout (HLTF-KO). Right panel, HLTF RING domain point mutant (C760S) generated by CRISPR-Cas9. Control cells were untreated or treated with 1 μg/ml APH for 24 h. HLTF knockdown/ KO / KI cells were treated with APH for 24 h, with Chk1 inhibitor added during the final 4 h. Cells were harvested immediately after treatment. Tubulin served as a loading control.

(**C**) Clonogenic survival of control, HLTF-KO, and HLTF-RING domain point mutant (C760S) eHAP cells left untreated or treated with APH/Chk1i. Left panel, representative images of crystal violet–stained colonies. Right panel, quantification of colony numbers expressed as fold change relative to the untreated condition. Error bars represent the standard error of the mean (SEM).
(D) SHPRH, a human orthologe of yeast Rad5 like HLTF, was individually knocked out in U2OS cells.
(**E,F**) DNA fiber analysis of replication fork progression SHPRH KO U2OS cells. (**E**) Experimental set up. (**F**) Box-and-whisker plot and median IdU track length in bicolour fibers. At least 150 forks were scored per sample.
(**G**) Immunoblot. DNA damage was monitored by immunoblotting of pKAP1, pCHK1-S345, pRPA-S4/S8, and γH2AX in cells treated with APH +/- Chk1 inhibitor. Bottom panel, quantification of γH2AX intensity from the blot.
(H) Immunoblot. ZRANB3 and SMARCAL1 were knocked out in U2OS cells, individually or in combination.
(**I**,**J**) DNA fiber analysis of replication fork progression in single or double ZRANB3 and SMARCAL1 knockout U2OS cells. (**I**) Experimental set up. (**J**) Box-and-whisker plot and median IdU track length in bicolour fibers. At least 150 forks were scored per sample.
(**K**) Immunoblot. DNA damage was monitored by immunoblotting of pKAP1, pCHK1-S345, pRPA-S4/S8, and γH2AX in cells treated with APH +/- Chk1 inhibitor. Bottom panel, quantification of γH2AX intensity from the blot.
(L) Immunofluorescence analysis of DNA damage in HLTF, ZRANB3 or SMARCAL1 knockout cells treated with APH, with or without Chk1 inhibitor. DNA damage was assessed by detection of chromatin-bound RPA32 and parental single-stranded DNA (P-ssDNA). Upper panel, representative images. Bottom panel, quantifications.
(M) HLTF, ZRANB3, and SMARCAL1 knockout cells were treated with control siRNA or siRNAs

targeting HLTF, ZRANB3, or SMARCAL1, as indicated, and exposed to APH with or without Chk1 inhibitor. DNA damage was assessed by immunoblotting for pCHK1-S345, pRPA-S4/S8, and γH2AX. Right panel, quantification of γH2AX signal intensity from the blot.

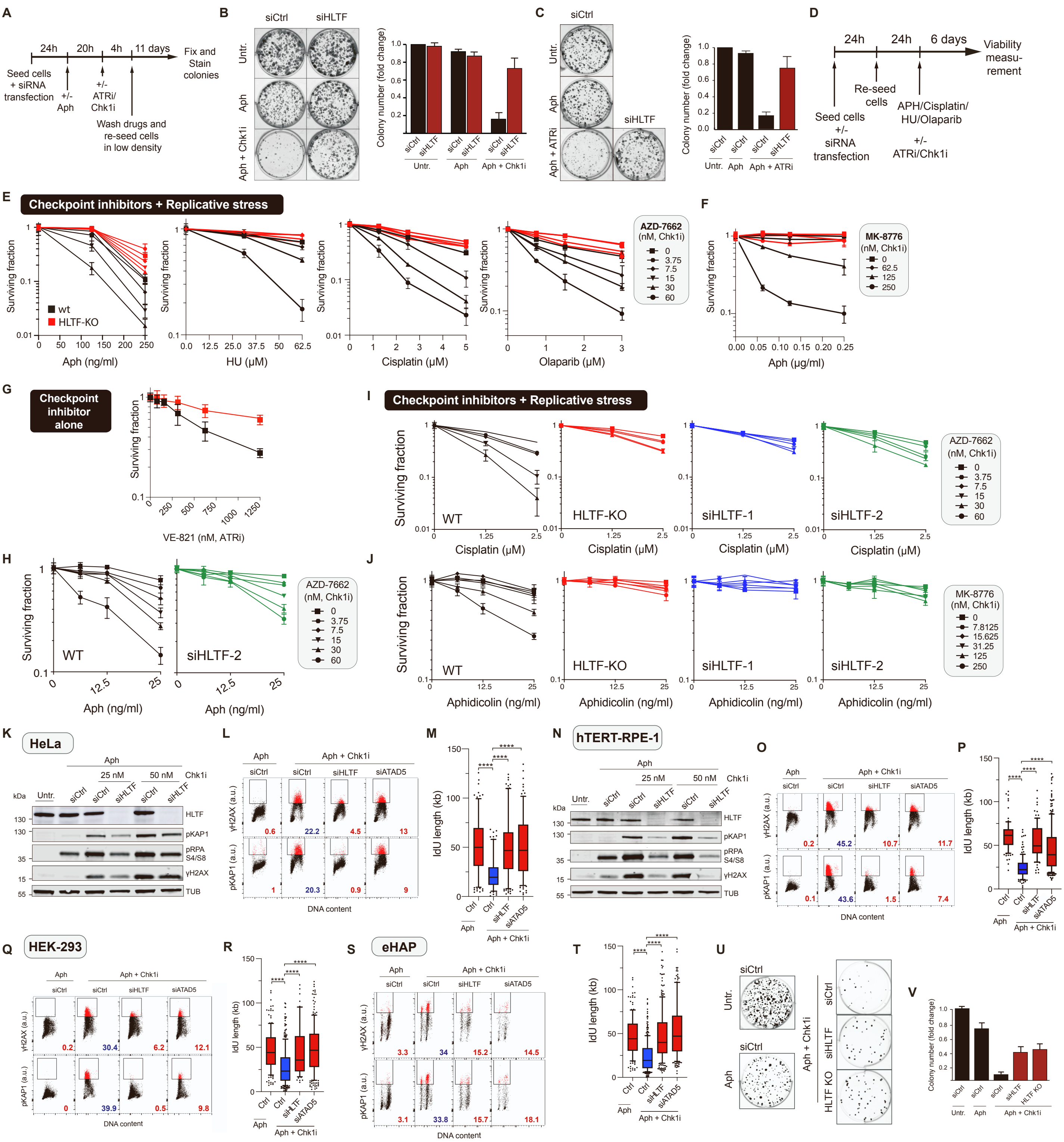

Figure S6. HLTF is responsible for the defects caused after checkpoint inhibition, related to Figure 4.

(A–C) Clonogenic survival analysis following HLTF depletion in U2OS cells.**(A)** Schematic of the colony formation assay workflow.**(B–C)** U2OS cells treated with Chk1i (AZD7662, 50 nM) (**B**) or ATRi (VE-822, 500 nM) (**C**). Left panel, representative images of crystal violet–stained colonies.

Right panel, quantification of colony numbers, expressed as fold change relative to untreated controls. Error bars indicate standard error of the mean (SEM).

(D–J) Cell viability assays of HLTF-KO and HLTF-depleted U2OS cells using CellTiter-Glo. **(D)** Schematic of the CellTiter-Glo viability assay setup. **(E–G)** HLTF-KO U2OS cells were treated with increasing concentrations of Chk1 inhibitors (AZD7662, **E**; MK-8776, **F**) or ATR inhibitor (VE-821, **G**) either alone or in combination with genotoxic agents (APH, HU, cisplatin, or olaparib) at the indicated concentrations. **(H–J)** Survival analysis of HLTF-KO and HLTF-depleted U2OS cells using two different siRNAs (siHLTF-1 and siHLTF-2). **(H)** HLTF-depleted (siHLTF-2) U2OS cells treated with APH and increasing concentrations of Chk1i, related to **Figure 4G**. **(I–J)** Cells were treated with Chk1 inhibitors (AZD7662, **I**; MK-8776, **J**) alone or in combination with genotoxic agents (cisplatin, **I**; APH, **J**). For all assays, viability was normalised to cells treated with the checkpoint inhibitor alone (without genotoxic agents).

(K–U) Impact of HLTF and ATAD5 depletion after checkpoint inhibition in various human cell lines. **(K–M)** HeLa (cervical cancer); **(N–P)** RPE1 (non-transformed, telomerase-immortalised retinal epithelial); **(Q–R)** HEK-293 (non-cancerous, adenovirus-transformed embryonic kidney); **(S–V)** eHAP (haploid chronic leukemia). **(K, N)** DNA damage assessed by immunoblotting. **(L, O, Q, S)** Quantification of DNA damage markers by flow cytometry after 20 h APH treatment alone or combined with 4 h checkpoint inhibition. **(M, P, R, T)** Replication fork progression by DNA fiber assay. Cells were sequentially labelled with CldU (20 min), treated with IdU + APH (1 µg/ml) ± Chk1i (AZD7662, 50 nM) for 4 h, then pulsed with IdU alone (40 min). Data shown as box-and-whisker plots with median IdU track length in bicolour fibers; ≥125 forks analysed per condition. **(U, V)** Clonogenic survival of HLTF-KO and HLTF-depleted eHAP cells treated with Chk1i (AZD7662, 50 nM). Left panel, representative crystal violet–stained colonies. Right panel, colony number shown as fold change relative to untreated controls.
